# Splicing factor hnRNPA1 regulates alternative splicing of *LOXL2* to enhance the production of *LOXL2Δ13*

**DOI:** 10.1016/j.jbc.2024.107414

**Published:** 2024-05-27

**Authors:** Deyuan Pan, Lin Long, Chengyu Li, Yingxin Zhou, Qing Liu, Ziting Zhao, Hui Zhao, Wan Lin, Zhenyuan Zheng, Liu Peng, Enmin Li, Liyan Xu

**Affiliations:** 1 Key Laboratory of Molecular Biology for High Cancer Incidence Coastal Chaoshan Area, Department of Biochemistry and Molecular Biology, Shantou University Medical College, Shantou, Guangdong Province, China; 2 State Key Laboratory of Pathogenesis, Prevention and Treatment of High Incidence Diseases in Central Asia, Xinjiang Medical University, Urumqi, China; 3 Institute of Basic Medical Science, Cancer Research Center, Shantou University Medical College, Shantou, Guangdong Province, China; 4 Institute of Oncologic Pathology, Shantou University Medical College, Shantou, Guangdong Province, China

**Keywords:** LOXL2, *LOXL2Δ13*, hnRNPA1, alternative splicing, exon skipping

## Abstract

Lysyl oxidase-like 2 (LOXL2) is a member of the lysyl oxidase family and has the ability to catalyze the cross-linking of extracellular matrix collagen and elastin. High expression of LOXL2 is related to tumor cell proliferation, invasion, and metastasis. *LOXL2* contains 14 exons. Previous studies have found that *LOXL2* has abnormal alternative splicing and exon skipping in a variety of tissues and cells, resulting in a new alternatively spliced isoform denoted *LOXL2Δ13*. *LOXL2Δ13* lacks *LOXL2WT* exon 13, but its encoded protein has greater ability to induce tumor cell proliferation, invasion, and metastasis. However, the molecular events that produce *LOXL2Δ13* are still unclear. In this study, we found that overexpression of the splicing factor hnRNPA1 in cells can regulate the alternative splicing of *LOXL2* and increase the expression of *LOXL2Δ13*. The exonic splicing silencer exists at the 3′ splice site and 5′ splice site of *LOXL2* exon 13. HnRNPA1 can bind to the exonic splicing silencer and inhibit the inclusion of exon 13. The RRM domain of hnRNPA1 and phosphorylation of hnRNPA1 at S91 and S95 are important for the regulation of *LOXL2* alternative splicing. These results show that hnRNPA1 is a splicing factor that enhances the production of *LOXL2Δ13*.

Lysyl oxidase-like 2 (LOXL2) is a member of the lysyl oxidase (LOX) family. The full-length *LOXL2* contains 14 exons and encodes a protein of molecular weight 86.7 kD. LOXL2 has LOX activity and catalyzes the cross-linking of elastin and collagen in the extracellular matrix (1, 2). Studies have shown, in a variety of cancers, that increased expression of LOXL2 is related to epithelial-mesenchymal transition, tumor development, invasion, and metastasis and is a marker of epithelial-mesenchymal transition and poor prognosis (3, 4, 5). *LOXL2* pre-mRNA will produce many isoforms during splicing (6, 7, 8). *LOXL2Δ13* (KF928961) is an alternatively-spliced isoform of *LOXL2* found by our laboratory in 2014 (6). Compared with *LOXL2WT*, *LOXL2Δ13* lacks exon 13 due to exon skipping, resulting in a shortened C-terminal LOX domain of the translated protein. Previous studies found that *LOXL2Δ13* is expressed in many cell lines and esophageal cancer tissues, but the expression level of *LOXL2Δ13* in cells is generally lower than that of *LOXL2WT* (6). Although the LOX region of LOXL2Δ13 is shortened and its deamination activity is partially reduced, LOXL2Δ13 is more strongly transforming in esophageal squamous cell carcinoma cells, compared to LOXL2WT, indicating that the cancer-promoting function of LOXL2Δ13 does not depend on LOX activity (6).

The cancer-promoting function of LOXL2WT or LOXL2Δ13 is related to rearrangement of the cytoskeleton and metabolic reprogramming (9, 10). In the case of cytoskeletal rearrangement, LOXL2WT or LOXL2Δ13 may promote the invasion and metastasis of esophageal cancer by enhancing the phosphorylation of ezrin T567 by PKC (9). Overexpression of LOXL2WT or LOXL2Δ13 can promote the proliferation of esophageal cancer cells. Both LOXL2WT and LOXL2Δ13 can directly catalyze the deacetylation of aldolase A K13, releasing aldolase A from the cytoskeleton, enhancing both the enzymatic activity of aldolase A and glycolysis of tumor cells, regulate metabolic reprogramming, and promote tumor development (10). LOXL2Δ13 is also related to obesity, which can affect the homeostasis of intestinal microflora and lipid metabolism and inhibit adipose tissue differentiation by inhibiting adipose tissue differentiation-related genes, leading to fat loss and reduced obesity (11). These results show that LOXL2Δ13 plays an important role in the occurrence and development of tumors and in reducing obesity. However, the mechanism of regulating *LOXL2* exon 13 skipping is still unclear.

Alternative splicing (AS) of pre-mRNA is an important mechanism for producing proteomic diversity in eukaryotes and also an important regulatory method for cell proliferation, differentiation, apoptosis, and ontogenesis (12). More than 90% of genes in eukaryotes undergo AS. Pre-mRNA generates various mature mRNAs by connecting different splice sites during the splicing process. These differently-spliced mRNA isoforms produced by transcription of the same gene will be translated into proteins with different functions (13). *Cis*-acting elements and *trans*-regulators are two major factors that regulate AS of pre-mRNA. *Cis*-acting elements refer to specific sequences on the pre-mRNA, including the 5′ splice site (5′ SS), 3′ splice site (3′ SS), branch point (BP), splicing enhancers, and splicing silencers. *Cis*-elements located on exons include exonic splicing enhancer and exonic splicing silencer (ESS). *Cis*-elements located on introns include intron splicing enhancer and intron splicing silencer (ISS). *Trans*-regulatory factors are mainly splicing factors, including the serine/arginine-rich protein (SR) family (14) and heterogeneous nuclear ribonucleoprotein (hnRNP) family (15), as well as other splicing factors, such as CELF, MBNL, RBFOX, STAR, and NOVA (16). During AS, SR family proteins usually bind an exonic splicing enhancer to promote the recognition of specific splice sites by the spliceosome, thereby increasing the occurrence of splicing reactions. However, hnRNP family proteins generally bind ESSs and ISSs to inhibit the splicing reaction. In general, a gene contains multiple *cis*-acting elements that recruit corresponding splicing factors. There is a mutual promotion or competition between them, forming a complex network that regulates AS (17, 18).

Heteronuclear ribonucleoprotein A1 (hnRNPA1) is a member of the hnRNP splicing factor family. hnRNPA1 is highly expressed in cells and is one of the most abundant core proteins in the hnRNP family (19). HnRNPA1 is a multifunctional protein that plays an important role in AS and transport of RNA and protects telomere DNA (20). HnRNPA1 is a typical splicing silencer and can combine with ESS or ISS to inhibit RNA splicing. For example, hnRNPA1 can recognize the UAGGGC sequence to bind the 5′ SS of pyruvate kinase M1/2 (*PKM*) intron 9, blocking the binding of U1 snRNP to the 5′ SS, and inhibiting the splicing of exon 9, producing *PKM2* mRNA containing exon 10, and inhibiting the generation of *PKM1* mRNA containing exon 9 (21, 22, 23, 24). There are a variety of posttranslational modifications of hnRNPA1, such as phosphorylation, ubiquitination, SUMOylation, methylation, and acetylation. Among them, there are many phosphorylation sites, mainly on serines. Phosphorylated hnRNPA1 will change its subcellular localization, causing accumulation in the cytoplasm and reducing its ability to bind RNA (25, 26). In this paper, we investigate the splicing factor that promotes the production of *LOXL2Δ13* and the effect of phosphorylated hnRNPA1 on the AS of *LOXL2*. We find that S91/S95-unphosphorylated hnRNPA1 binds the 3′ SS and 5′ SS of *LOXL2* exon 13 to regulate the splicing of exon 13 and enhance the production of *LOXL2Δ13*.

## Results

### Multiple splicing factors bind in the region of *LOXL2* exon 13 and regulate its splicing

**Figure 1:**
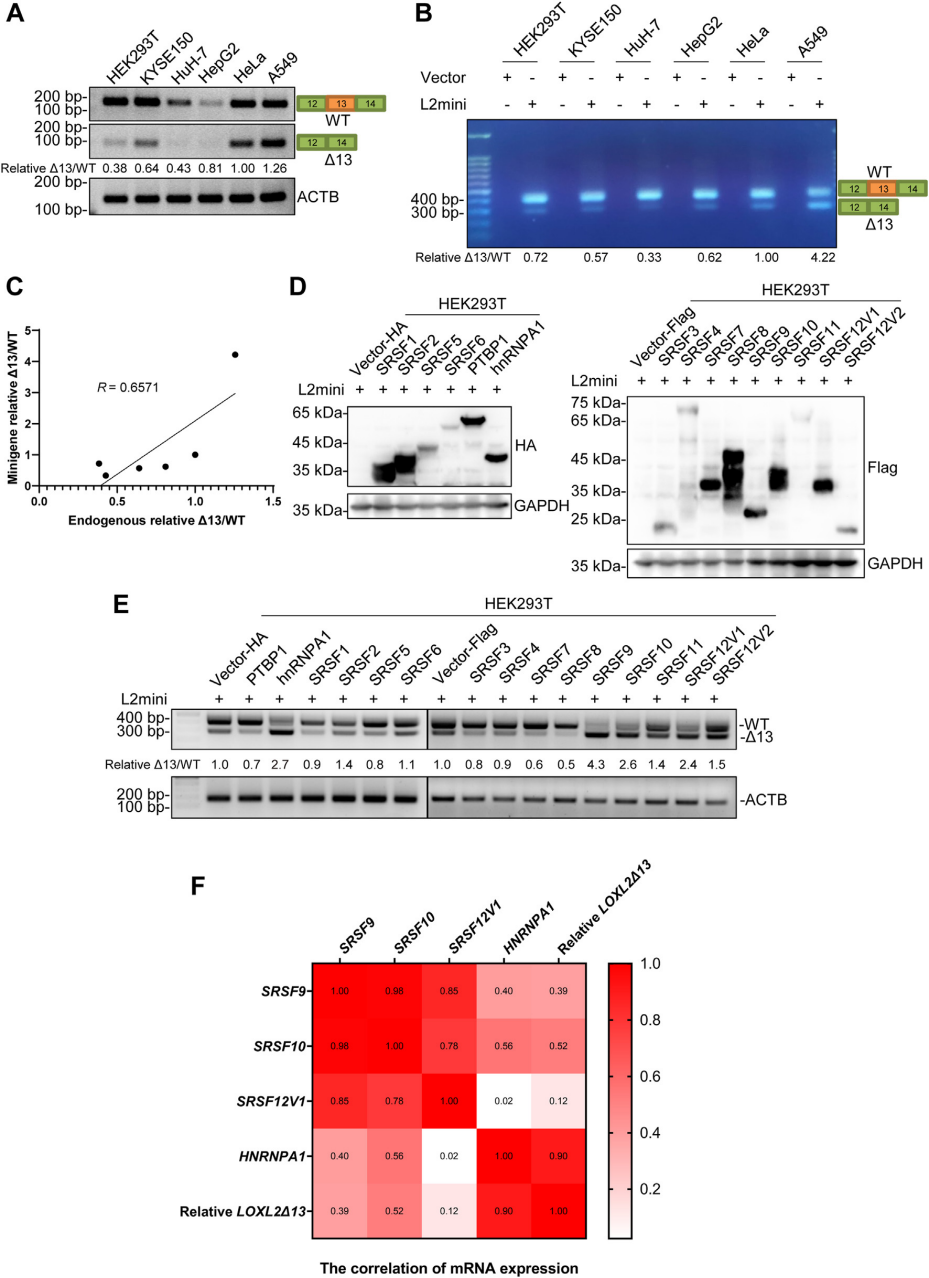
**Multiple splicing factors bind in proximity to *LOXL2* exon 13 and regulate its splicing.***A*, RT-PCR analysis of the splicing of the endogenous *LOXL2* in HEK293T, KYSE150, HuH-7, HepG2, HeLa, and A549 cells. The *upper* band represents *LOXL2WT* and the *lower* band represents *LOXL2Δ13*. Relative Δ13/WT refers to the ratio of *LOXL2Δ13* to *LOXL2WT*, with HeLa as the benchmark. *B*, RT-PCR analysis of the splicing of the *LOXL2* minigene in HEK293T, KYSE150, HuH-7, HepG2, HeLa, and A549 cells. The *upper* band represents *LOXL2WT* and the *lower* band represents *LOXL2Δ13*. Relative Δ13/WT ratios were calculated. *C*, Spearman correlation was used to analyze the correlation between endogenous relative Δ13/WT and minigene relative Δ13/WT. *R* = 0.6571, *p* = 0.175. *D*, Western blot analysis of the overexpression of splicing factors SRs 1-12, PTBP1, and hnRNPA1 in HEK293T and HeLa cells. *E*, splicing of the *LOXL2* minigene regulated by splicing factors was analyzed by RT-PCR. Cotransfection of individual splicing factors with the *LOXL2* minigene in HEK293T cells. Relative Δ13/WT represents the ratio of band *gray* values between *LOXL2Δ13* and *LOXL2WT*. ACTB was used as an internal control for sample loading. *F*, RT-qPCR was used to detect the RNA levels of hnRNPA1, SRSF9, SRSF10, SRSF12V1, *LOXL2WT*, and *LOXL2Δ13* in six cell lines. Pearson correlation was used to analyze the correlation and obtain a correlation heatmap.

In previous studies, we found that *LOXL2WT* and *LOXL2Δ13* are expressed in various cell lines and esophageal cancer tissues, and the expression of *LOXL2Δ13* in cells is generally lower than that of *LOXL2WT* (6). We questioned whether there are cells that express *LOXL2Δ13* at higher levels than *LOXL2WT*. So, we used RT-PCR to determine the RNA expression levels of *LOXL2WT* and *LOXL2Δ13* in the esophageal squamous cell carcinoma cell line KYSE150, human liver cancer cell lines HuH-7 and HepG2, human non-small cell lung cancer cell line A549, cervical cancer cell line HeLa, and human embryonic kidney cell line HEK293T. We found that the expression levels of *LOXL2WT* and *LOXL2Δ13* were very low in HepG2 and HuH-7 cells, whereas the RNA expression level was high in all other cell lines. However, the level of *LOXL2WT* RNA was still higher than that of *LOXL2Δ13* in all cell lines tested (Fig. 1*A*).

In order to further study the molecular mechanism of *LOXL2Δ13* production, we constructed a *LOXL2* minigene, using pcDNA3 as the expression vector and the CMV promoter to express the *LOXL2* minigene sequence containing complete exons 12-14 and intervening introns 12 and 13 (Fig. S1*A*). We verified the minigene *in vivo* splicing by transfecting the *LOXL2* minigene into the same six cell lines for expression and further confirmed the splicing out of exon 13, which occurs in the formation of *LOXL2Δ13*. RT-PCR showed that *LOXL2* minigene splicing resulted in two bands with a size difference of about 100 bp, consistent with our prediction of a *LOXL2Δ13* splicing event, known as an exon skipping. These two bands were sequenced and showed that the larger band was *LOXL2WT* and the smaller band was *LOXL2Δ13*, exactly missing the entire 112-bp exon 13 (Fig. S1*A*). We also found that the larger *LOXL2WT* band was brighter than the *LOXL2Δ13* band among all detected cell lines (Fig. 1*B*), indicating that the splicing of the *LOXL2* minigene in all detected cell lines was consistent with the splicing of endogenous *LOXL2* pre-mRNA, where the RNA expression level of *LOXL2WT* was significantly higher than that of *LOXL2Δ13*. Although the relative Δ13/WT values of the two are not consistent, there is a certain correlation (Fig. 1, *A*–*C*). These results indicate that the *LOXL2* minigene can reflect the splicing status of endogenous *LOXL2WT* and *LOXL2Δ13*.

Splicing factors are the main factors that govern the AS of pre-mRNA. By binding to *cis*-acting elements at different positions, splicing factors can promote or inhibit splicing reactions. We used two databases, ESEfinder 3.0 and SpliceAid, to predict splicing factors that bind near exon 13 of *LOXL2* pre-mRNA (27, 28). We found that there were a large number of RNA-binding proteins that could bind to the *LOXL2* exon 13, including two major splicing factors involving the SR family (SFSR1, SFSR2, SFSR3, SFSR5, SFSR6, and SFSR9), as well as the hnRNP family (PTBP1 and hnRNPA1) (Fig. S1*B*). The specific binding sites identified by these splicing factors have been identified (17). It has been reported that PTBP1, as a pyrimidine tract–binding protein, can bind to pyrimidine sequences upstream of the exon, and hnRNPA1 can recognize the UAG sequence, and both of these splicing factors can inhibit exon splicing (22).

To identify splicing factors that could regulate AS of *LOXL2*, we constructed plasmids expressing SR proteins SRSF1 to SRSF12, as well as PTBP1 and hnRNPA1 of the hnRNP family (Figs. 1*D* and S2*A*). We cotransfected the various splicing factor plasmids with the *LOXL2* minigene into HEK293T and HeLa cells to analyze the effects of various splicing factors on the AS of *LOXL2*. We found that most of the splicing factors could affect the splicing of the *LOXL2* minigene (Figs. 1*E* and S2*B*). Among them, hnRNPA1, SRSF9, SRSF10, and SRSF12 could promote the production of minigene-*LOXL2Δ13* (relative Δ13/WT ≥ 1.5). Next, we characterized the RNA expression levels of hnRNPA1, SRSF9, SRSF10, *SRSF12V1*, *LOXL2WT*, and *LOXL2Δ13* in six cell lines (Fig. S4). By analyzing the correlation, we found that only hnRNPA1 was positively correlated with relative *LOXL2Δ13* (Fig. 1*F*), indicating that when hnRNPA1 expression is increased, the expression of *LOXL2Δ13* is also increased compared to *LOXL2WT*. Therefore, we focused on hnRNPA1 as the main research object.

### Expression of hnRNPA1 increases the production of *LOXL2Δ13*

**Figure 2:**
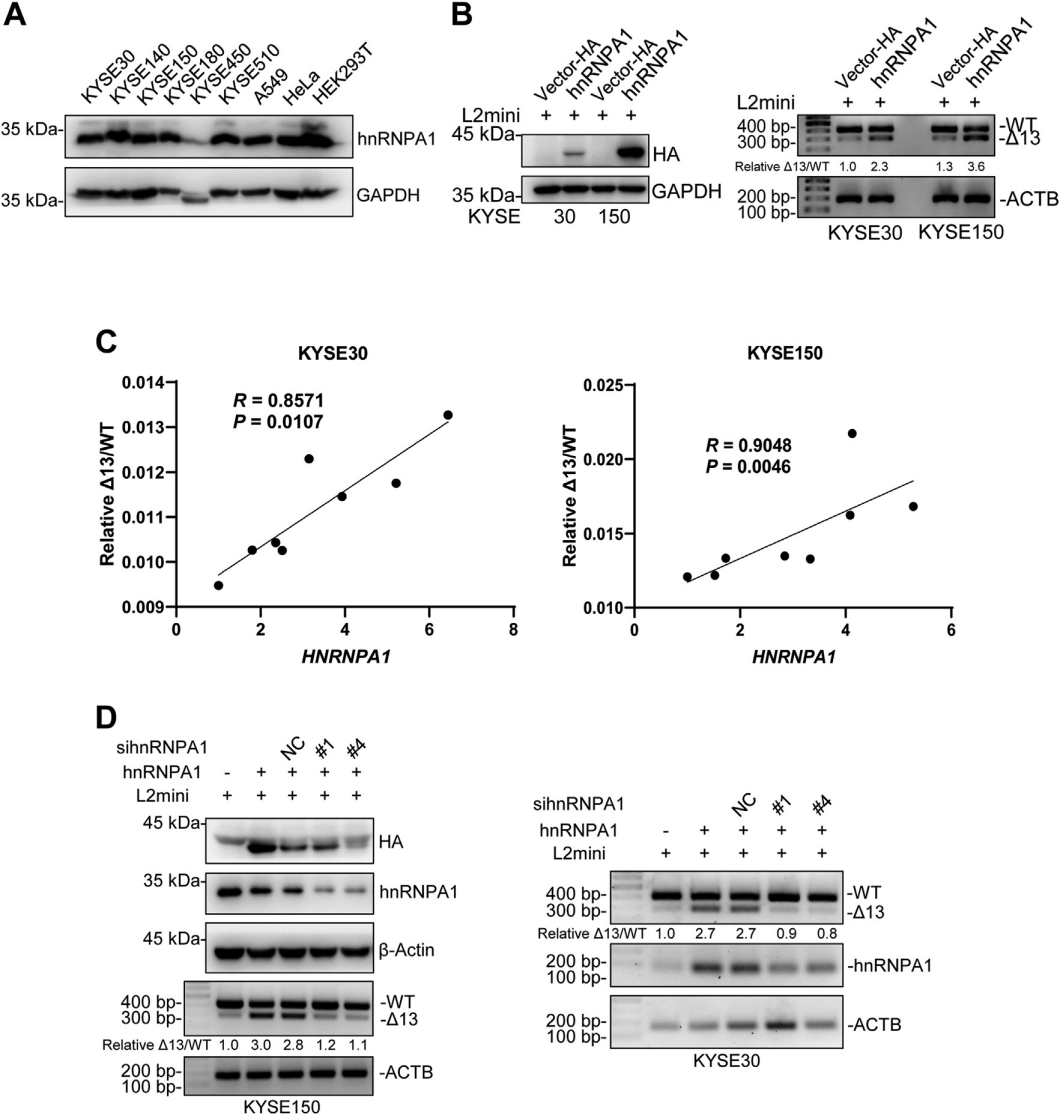
**Expression of hnRNPA1 affects the expression of *LOXL2Δ13*.***A*, expression of endogenous hnRNPA1 in different cell lines. *B*, overexpression of hnRNPA1 in KYSE30 and KYSE150 cells regulated *LOXL2* minigene splicing. Expression of hnRNPA1 was detected by Western blotting. Relative Δ13/WT ratios were calculated. *C*, RT-qPCR was used to detect the RNA levels of *HNRNPA1*, *LOXL2WT*, and *LOXL2Δ13* in KYSE30 and KYSE150 cells. HnRNPA1 was transfected into cells (transfected plasmid amounts: 0.6, 0.8, 1.0, 1.2, 1.4, 1.6, 1.8 μg). Spearman correlation was used to analyze the correlation between *hnRNPA1* and endogenous relative Δ13/WT. *D*, effect of KYSE150 and KYSE30 overexpression and knockdown of hnRNPA1 on *LOXL2* minigene splicing. The first and second lanes are the vector and hnRNPA1-HA cotransfected with the *LOXL2* minigene, respectively, and the third to fifth lanes show hnRNPA1-HA, siRNA, and *LOXL2* minigene cotransfections. Relative Δ13/WT ratios were calculated.

We determined the protein expression level of hnRNPA1 in nine cell lines and found that the protein expression levels of hnRNPA1 in the different cell lines were high (Fig. 2*A*). In the proteomics database of 124 patients with esophageal cancer (29), tumors showed higher hnRNPA1 expression than normal tissue (Fig. S2*C*). We cotransfected plasmids expressing hnRNPA1 and the *LOXL2* minigene into KYSE30, KYSE150, and A549 cells. RT-PCR analysis showed that minigene splicing was consistent with the above results showing the expression of minigene-*LOXL2WT* decreased and the expression of minigene-*LOXL2Δ13* increased (Figs. 2*B* and S2*D*). We also transfected hnRNPA1 in KYSE30 and KYSE150 cells and determined a positive correlation between endogenous relative Δ13/WT and hnRNPA1 expression through qPCR (Fig. 2*C*). After that, by simply knocking down endogenous hnRNPA1 in KYSE150 and A549 cells and cotransfecting the *LOXL2* minigene, the expression of minigene-*LOXL2Δ13* only slightly decreased (Fig. S2*E*), possibly because the expression level of *LOXL2Δ13* was already inherently low and the effect of knocking down hnRNPA1 to decrease in *LOXL2Δ13* would not be significant. To solve this problem, we cotransfected hnRNPA1, the *LOXL2* minigene, and siRNA for hnRNPA1 into KYSE30 and KYSE150 cells. We found that when only hnRNPA1 and the minigene were transfected, the expression of minigene-*LOXL2WT* decreased and the expression of minigene-*LOXL2Δ13* increased compared with the vector control. When hnRNPA1, the *LOXL2* minigene, and the siRNA for hnRNPA1 were cotransfected, hnRNPA1 overexpression first increased the expression of minigene-*LOXL2Δ13*, followed by the downregulation of minigene-*LOXL2Δ13* and upregulation of minigene-*LOXL2WT* due to the ensuing effects of siRNA-mediated knock down of endogenous and exogenous hnRNPA1 (Fig. 2*D*). In this way, the difference in minigene-*LOXL2Δ13* expression was very significant. The decreased expression level of hnRNPA1 could indeed inhibit the expression of minigene-*LOXL2Δ13*. Therefore, the above results indicate that hnRNPA1 can increase the expression of *LOXL2Δ13* (both minigene and endogenous) by regulating the AS of *LOXL2* pre-mRNA.

### HnRNPA1 recognizes the UAG sequence in *LOXL2* exon 13 and binds to the 3′ SS and 5′ SS

**Figure 3:**
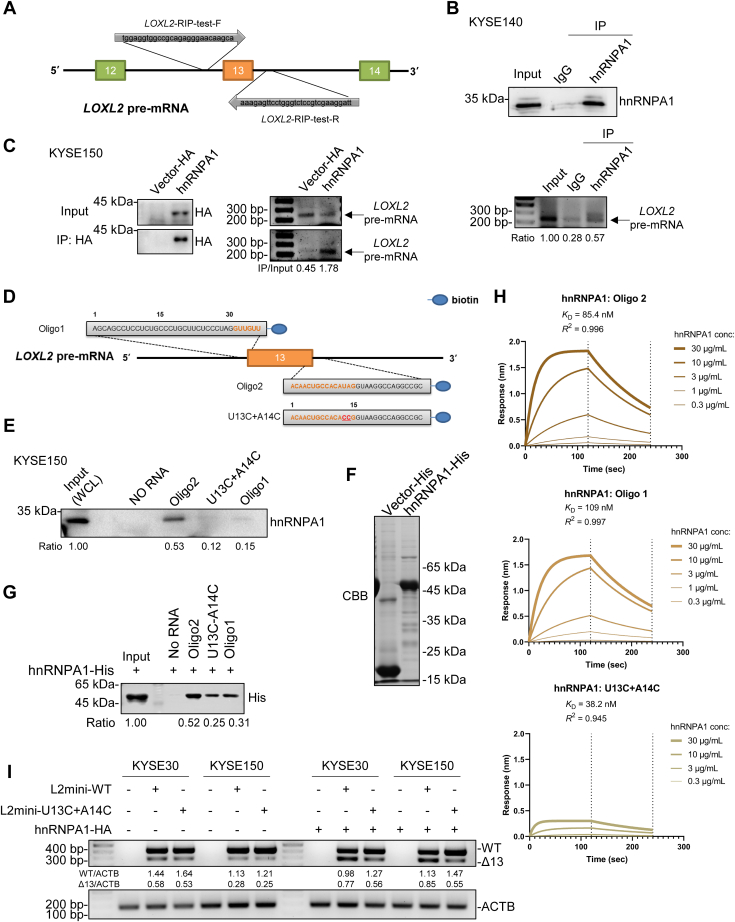
**HnRNPA1 recognizes and binds to the UAG sequence of the 3′ SS and 5′ SS of *LOXL2* exon 13.***A*, schematic diagram of RIP-PCR. *LOXL2*-RIP-test-F/R were the forward and reverse primers for detection. *B*, RIP test results of KYSE140 cells were used to detect the binding of endogenous hnRNPA1 and *LOXL2* pre-mRNA. The band indicated by the *arrow* is *LOXL2* pre-mRNA. Ratio refers to IP/Input, with input as the benchmark. *C*, RIP test results of KYSE150 cells were used to detect the binding of exogenous hnRNPA1 and *LOXL2* pre-mRNA. The band indicated by the *arrow* is *LOXL2* pre-mRNA. *D*, schematic diagram of the biotin-labeled RNA probe. The *black* sequences are located in the intron, the *orange* sequences are located in the exon, and the *red* sequence indicates the mutation site. *E*, RNA-pulldown was carried out in KYSE150 cells to verify the binding site of hnRNPA1. Ratio refers to pull-down/input, with input as the benchmark. *F*, coomassie bright blue result of purified hnRNPA1-His. *G*, Western blot showing the pull-down results of hnRNPA1-His with oligo1, oligo2, and U13C+A14C. Ratio represents pull-down/input. *H*, biolayer interferometry was used to calculate the kinetic characterization among hnRNPA1, oligo1, oligo2, and U13C+A14C. Streptavidin probes are used to bind biotin-RNA. *I*, effects of the overexpression of hnRNPA1 on the splicing of the vector (pcDNA3), WT, and U13C+A14C mutant *LOXL2* minigenes in cells. The experiment was carried out in KYSE30 and KYSE150 cells, which were divided into a control group and overexpression group. 5′ SS, 5′ splice site; 3′ SS, 3′ splice site.

The above results show that hnRNPA1 can regulate the AS of *LOXL2* pre-mRNA. Next, we further studied the mechanism by which hnRNPA1 regulates the splicing of *LOXL2* exon 13. HnRNPA1 is a typical splicing factor that can bind to ESS and inhibit the splicing of exons. Previous studies have shown that hnRNPA1 is able to recognize and bind UAGACA in *SMN2* exon 7 and UAGGGC in *PKM* exon 9, and UAGGGA/U is a high-affinity hnRNPA1-binding sequence, as determined by SELEX (22, 30, 31). These reported hnRNPA1-binding motifs mostly contain UAG sequences. Interestingly, by analyzing the sequence of *LOXL2* pre-mRNA, we found that out of all 14 exons of *LOXL2*, only the 5′ SS and 3′ SS of exon 13 and the 5′ SS of exon 10 contained UAG sequences, while none of the other exons had a UAG sequence near the 3′ SS and 5′ SS (Fig. S3*A*). So we speculated that hnRNPA1 was likely to bind to the 3′ SS and 5′ SS of exon 13 to regulate the AS of the *LOXL2* pre-mRNA. First, we wanted to know if hnRNPA1 could bind to *LOXL2* pre-mRNA. We found that hnRNPA1 could indeed bind to the pre-mRNA of *LOXL2* through RNA immunoprecipitation (RIP) assays (Fig. 3, *A*–*C*). We expressed hnRNPA1 in HeLa and KYSE150 cells, extracted the total protein, and then performed RNA-pulldown on the total protein using biotinylated oligonucleotide oligo1 from the 3′ SS and biotinylated oligonucleotide oligo2 from the 5′ SS (Fig. 3*D*). The results indicate that hnRNPA1 can bind to the 3′ SS and 5′ SS of *LOXL2* exon 13, with hnRNPA1 being more capable of binding to 5′ SS (Figs. 3*E* and S3*B*). To determine the binding sequence of hnRNPA1, we mutated the UAG of oligo2, which had displayed stronger binding, into CCG (U13C + A14C). The RNA-pulldown results of U13C+A14C indicate that the mutated oligo2 bound very little hnRNPA1 (Figs. 3*E* and S3*B*). Then we purified His-tagged hnRNPA1 and used oligo1, oligo2, and U14C+A14C to pull down hnRNPA1-His. The results were consistent with previous findings, demonstrating that hnRNPA1 can directly bind to the 3′ SS and 5′ SS of *LOXL2* exon 13 (Fig. 3, *F* and *G*). We measured the affinity, between hnRNPA1 and oligo1, oligo2, and U13C+A14C, using biolayer interferometry (BLI). The results showed that compared with oligo1, oligo2 had a lower *K*_*D*_ and stronger affinity for hnRNPA1. Compared with oligo2, although U14C+A14C had a lower *K*_*D*_ and stronger affinity for hnRNPA1, it carried less hnRNPA1 (Fig. 3*H*). Thus, hnRNPA1 can directly bind to the 3′ SS and 5′ SS of *LOXL2* exon 13 by recognizing the UAG sequence and has a stronger affinity for the 5′ SS.

To investigate whether hnRNPA1 regulates *LOXL2* splicing by recognizing the UAG sequence of *LOXL2* exon 13, we constructed a *LOXL2* minigene with identical “U13C+A14C” mutations (Fig. 3*D*). Compared with the WT *LOXL2* minigene, the “U13C+A14C” mutant *LOXL2* minigene had increased *LOXL2WT* and decreased *LOXL2Δ13* expression (Figs. 3*I* and S3, *C* and *D*). Therefore, we can infer that the altered splicing of the mutant *LOXL2* minigene is caused by the abnormal binding of hnRNPA1 to the UAG at the 5′ SS of exon 13 of LOXL2. Similarly, hnRNPA1 can also bind to the UAG of the 3′ SS of *LOXL2* exon 13 to regulate splicing of *LOXL2* exon 13. To sum up, these *in vitro* experimental results indicate that hnRNPA1 regulates the splicing of *LOXL2* by directly binding to the 5′ SS and 3′ SS UAG sequences of *LOXL2* exon 13. It is highly likely that hnRNPA1 regulates the splicing of endogenous *LOXL2* exon 13 in this manner.

### RRM domain deletion inhibits the splicing regulatory function of hnRNPA1

**Figure 4:**
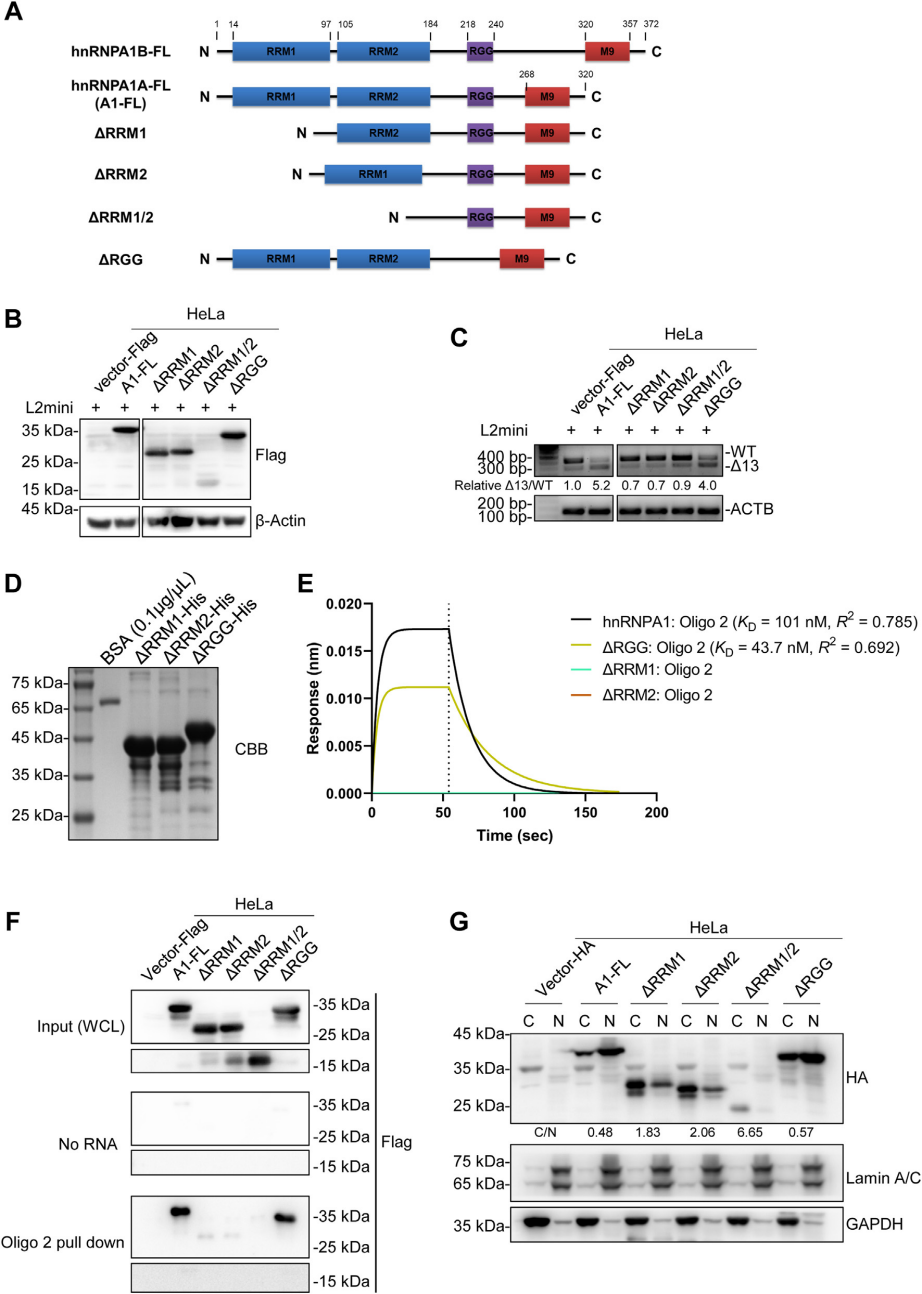
**Deletion of RRM domains affects the splicing function of hnRNPA1.***A*, structural diagram of hnRNPA1 full-length and deletion mutants. *B*, expression of hnRNPA1 full-length and deletion mutants in HeLa. pcDNA3.1-N-SBP-HA and pCMV-N-Flag served as vectors. *C*, RT-PCR was used to detect the effect of different deleted hnRNPA1 domains on hnRNPA1-mediated splicing. Relative Δ13/WT ratios were calculated. *D*, Coomassie brilliant blue staining of purified ΔRRM1-His, ΔRRM2-His, and ΔRGG-His. *E*, biolayer interferometry was used to calculate the kinetic characterization of hnRNPA1-His, ΔRRM1-His, ΔRRM2-His, and ΔRGG-His with oligo2. Streptavidin probes were used to bind biotin-RNA. HnRNPA1 without either RRM does not fit the binding curve. The figure shows the binding curves fitted by hnRNPA1-His and ΔRGG-His with oligo2. The *dashed* line represents a time of 54 s. *F*, RNA-pulldown was carried out in HeLa cells to verify the domain that regulates the binding of hnRNPA1 to RNA. *G*, determination of hnRNPA1 subcellular localization by HeLa cell fractionation. N represents the nuclear component, shown by lamin A/C antibody, C represents the cytoplasmic component, shown by GAPDH antibody. HA antibody was used to detect the overexpressed hnRNPA1. C/N represents the grayscale ratio of overexpressed hnRNPA1 in the cytoplasm and nucleus.

Next, we investigated the mechanism by which hnRNPA1 regulates the AS of *LOXL2* exon 13 from the perspective of structure. Previous studies show that hnRNPA1 mainly exists as two isoforms, hnRNPA1A and hnRNPA1B. HnRNPA1B is 52 aa longer than hnRNPA1A, but hnRNPA1A is 20 times more abundant than hnRNPA1B (19). The structure of hnRNPA1 mainly includes RNA recognition motifs RRM1 and RRM2, an RGG box rich in RGG sequences, and the nuclear localization sequence M9, as shown in Figure 4*A* (19). Compared to hnRNPA1A, hnRNPA1B has an additional 52 aa between the RGG box and M9 sequence. We cotransfected hnRNPA1B and the *LOXL2* minigene into HEK293T cells. The *in vivo* splicing results showed that hnRNPA1B, like hnRNPA1A, can promote the skipping of *LOXL2* exon 13 (Fig. S5*A*). Therefore, hnRNPA1A was mainly used for further study. It has been reported that hnRNPA1-mediated splicing mainly depends on RNA recognition motifs RRM1 and RRM2 (32). In order to study the influence of different hnRNPA1 domains on the splicing of *LOXL2*, we constructed hnRNPA1 deletion mutants lacking RRM1, RRM2, and RGG box sequences (Figs. 4*B* and S5*B*). These deletions, as well as full-length hnRNPA1, and the *LOXL2* minigene were cotransferred into HEK293T and HeLa cell lines, followed by RNA extraction and RT-PCR assays. The results showed that when one or both RRMs were absent, hnRNPA1 lost its splicing ability, whereas the absence of the RGG box had little effect (Figs. 4*C* and S5*C*), consistent with previous research findings (32).

Binding to pre-mRNA is an important basis for splicing factors to regulate splicing. Therefore, we determined the RNA-binding capability of various hnRNPA1 deletion mutants to biotinylated oligo2 by BLI. We found that hnRNPA1 without the RRM domains could not fit a binding curve, indicating that hnRNPA1 without RRM domains could barely bind to oligo2. However, hnRNPA1 lacking the RGG box could still bind to oligo2 (Figs. 4, *D* and *E* and S5*D*). We also performed RNA-pulldown. The results showed that hnRNPA1 lost its ability to bind RNA due to the absence of either RRM domain, while the absence of the RGG box had little effect on RNA binding (Fig. 4*F*). The splicing regulation by hnRNPA1 is also related to its subcellular localization. Previous studies have shown that the M9 motif is a nuclear localization sequence, and absence of the M9 motif seriously affects the nuclear localization of hnRNPA1. The absence of RRM domains may also affect hnRNPA1 nuclear translocation (32, 33, 34). By cell fractionation and immunofluorescence, we found that most hnRNPA1 was localized in the nucleus, but a small fraction existed in the cytoplasm. When the RRM domain was absent, there was less hnRNPA1 entering the nucleus, and more hnRNPA1 accumulating in the cytoplasm (Figs. 4*G* and S6). When the RGG box was absent, the nuclear localization of hnRNPA1 did not change much (Figs. 4*G* and S6). Splicing of pre-mRNA takes place in the nucleus. Deletion of the RRM domains in hnRNPA1 reduced nuclear import of hnRNPA1, resulting in failure to participate in the regulation of pre-mRNA splicing. Therefore, these results indicate that hnRNPA1 must contain two RRM domains in order to have complete RNA-binding capability and accurately localize in the nucleus to regulate the AS of pre-mRNA.

### Phosphorylation of S91 and S95 inhibits the splicing regulatory function of hnRNPA1

**Figure 5:**
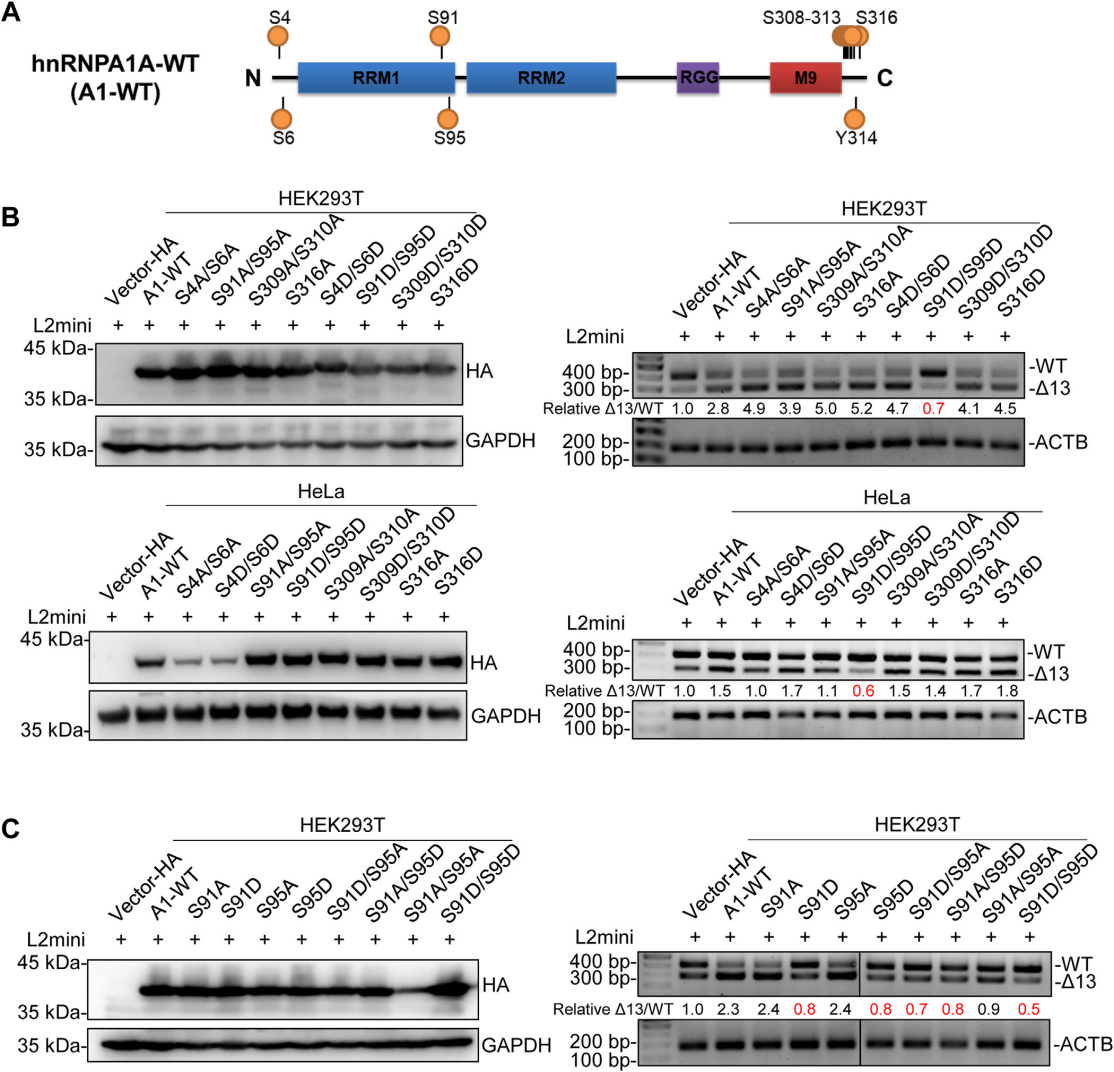
**Phosphorylation of hnRNPA1 S91 and 95 affects hnRNPA1-mediated splicing.***A*, schematic diagram of phosphorylated sites in hnRNPA1. *B* and *C*, effect of phosphorylation on splicing by hnRNPA1. Expression of hnRNPA1 phospho-mimetics and nonphosphorylatable mimetics (*left* side) and effects of hnRNPA1 phospho-mimetics and non-phosphorylatable mimetics on *LOXL2* minigene splicing (*right* side). Experiments were carried out in HEK293T and HeLa cells. Relative Δ13/WT ratios were calculated. *C*, effects of hnRNPA1 S91 and S95 mutations.

As with other splicing factors, hnRNPA1 has many posttranslational modifications, among which phosphorylation is the most abundant. Label-free phosphoproteomics for 31 pairs of tumor and nontumor esophageal tissues (29) showed twelve phosphorylation sites on hnRNPA1A to be distributed in the N-terminal, RRM, and C-terminal domains of hnRNPA1A (Fig. 5*A*). Most of these phosphorylation sites have been reported, but the phosphorylation modification of S91 seems to have not been reported (19, 35). In previous studies, the impact of phosphorylation on the function of hnRNPA1 was mainly studied in transcription and translation, while there was relatively little research on AS (35). Therefore, we investigated the effect of phosphorylation on the AS function of hnRNPA1. Among these twelve phosphorylation sites, only phosphorylation at S91 and S95 was downregulated in tumor esophageal tissues compared to nontumor esophageal tissues, while the rest were upregulated (Fig. S7). The phosphoproteomics data showed that there were eight phosphorylated modification sites at the C-terminus of hnRNPA1. Therefore, we chose three sites at the C-terminus, S309, S310, and S316, as well as four sites, S4, S6, S91, and S95 at the N-terminus, for further research. To investigate the role of phosphorylation at different sites of hnRNPA1 and their role in the AS of *LOXL2* exon 13, we constructed hnRNPA1 mutants that simulated phosphorylation and dephosphorylation (Fig. 5, *B* and *C*). Based on RT-PCR, we found that only the simulated phosphorylation of hnRNPA1 (S91D and S95D) reduced minigene-*LOXL2Δ13* levels compared to the WT hnRNPA1 (Fig. 5, *B* and *C*). For endogenous *LOXL2WT* and *LOXL2Δ13* in cells, the expression of WT and S91A/S95A mutant hnRNPA1 was positively correlated with the expression of *LOXL2Δ13*, while the correlation between S91D/S95D and *LOXL2Δ13* expression was reduced (Figs. 2*C* and S8). This indicates that the ability of hnRNPA1 to regulate splicing is reduced after S91 and S95 phosphorylation. The mutation from serine to alanine is a simulated nonphosphorylatable mutation that does not affect the splicing regulation function of hnRNPA1. This may be because the serine at this site itself exists in a nonphosphorylated state, so the serine mutation to alanine does not have much effect on its function.

**Figure 6:**
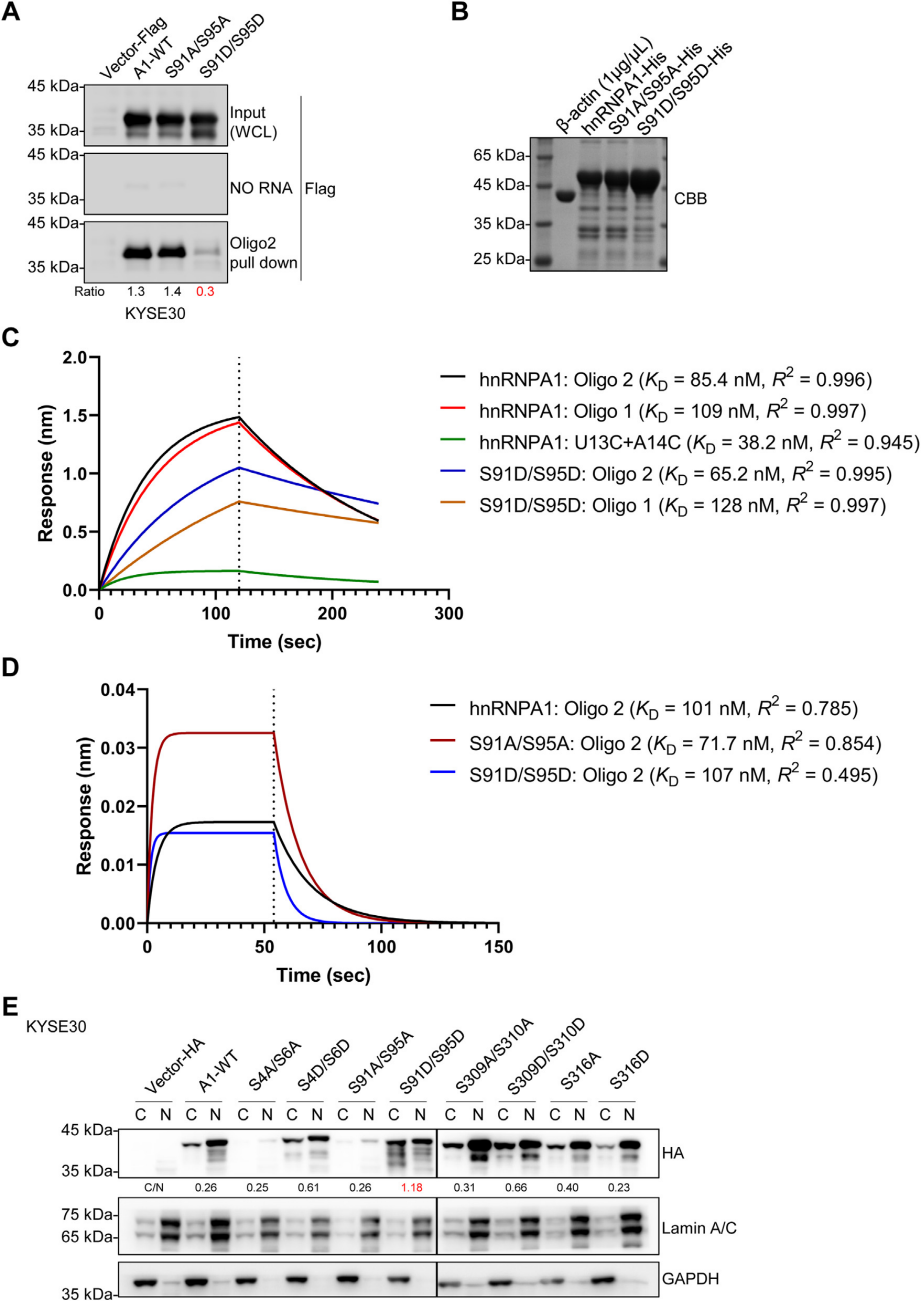
**Simulate phosphorylated hnRNPA1 (S91D) and (S95D) with reduced RNA-binding ability and reduced nuclear localization.***A*, RNA-pulldown was carried out in KYSE30 cells to determine the binding of simulated phosphorylation and simulated dephosphorylation of hnRNPA1 to RNA. Ratio refers to pull-down/input. *B*, Coomassie brilliant blue staining of purified hnRNPA1-His, S91A/S95A-His, and S91D/S95D-His. *C* and *D*, biolayer interferometry was used to calculate the kinetic characterization of hnRNPA1-His, S91A/S95A-His, and S91D/S95D-His with oligo1, oligo2, and U13C+A14C. *C*, streptavidin probes were used to bind biotin-RNA. *D*, Ni-NTA probes were used to bind hnRNPA1-His, S91A/S95A-His, and S91D/S95D-His. *E*, effect of phosphorylation on the subcellular localization of hnRNPA1, determined by cell fractionation of KYSE30 cells. N represents the nuclear component, characterized by lamin A/C antibody; C represents the cytoplasmic component, characterized by GAPDH antibody; HA antibody was used to characterize the overexpressed hnRNPA1. C/N represents the grayscale ratio of overexpressed hnRNPA1 in the cytoplasm and nucleus.

How does hnRNPA1 S91 and S95 phosphorylation regulate the splicing of *LOXL2*? Interestingly, we found that S91 and S95 happen to be located in RRM1, so we believe this may be related to RRM function. It has been reported that the binding of hnRNPA1 to RNA is also affected by phosphorylation (25). In addition, phosphorylation of hnRNPA1 can change its subcellular localization and cause its accumulation in the cytoplasm (26). By RNA-pulldown, we found that the ability of hnRNPA1 to bind RNA was lost due to the phosphorylation of S91 and S95 (Fig. 6*A* and S9*A*). Then, we purified hnRNPA1-His, S91A/S95A-His, and S91D/S95D-His (Fig. 6*B*) and further studied their RNA-binding ability through BLI. We conducted the experiment in two ways: we used a streptavidin probe-loaded biotinylated RNA to measure hnRNP1 binding or the Ni-NTA probe-loaded His-hnRNP1 to determine RNA binding. By conducting experiments with oligo2, we found that compared to WT hnRNPA1, the S91D/S95D had a similar or even stronger affinity for RNA but a reduced binding capacity (Fig. 6, *C* and *D*). This result is not consistent with the RNA-pulldown results of S91D/S95D on RNA. This may be due to the significant impact of S91- and S95-simulated phosphorylation mutations on the structure of hnRNPA1. There may also be some structural differences between S91D/S95D expressed in eukaryotic cells and S91D/S95D purified in prokaryotic cells. Therefore, there are differences in the binding patterns of these two S91D/S95D to RNA, but overall, these results show that the amount of S91D/S95D binding to RNA is lower than that of WT hnRNPA1. The affinity of S91A/S95A for RNA increases while the binding amount also increases (Fig. 6*D*). This result is consistent with the RNA-pulldown results of S91A/S95A expressed in KYSE30 and HEK293T cells (Figs. 6*A* and S9*A*). For oligo1, we found that these His-proteins did not fit the binding curve obtained through BLI (Fig. S9*B*). This is also consistent with previous results, indicating that hnRNPA1 binds more strongly to the 5′ SS of *LOXL2* exon13 than to the 3′ SS.

By immunofluorescence and cell fractionation assays, we found that phosphorylation of S91 and S95 led to the accumulation of hnRNPA1 in the cytoplasm, which increased cytoplasmic levels occurring with increasing phosphorylation (Figs. 6*E* and S10). These results suggest that phosphorylation of S91 and S95 in the RRM domain inhibited hnRNPA1-dependent splicing by altering the RNA-binding capacity and subcellular localization of hnRNPA1, thereby regulating the AS of *LOXL2* pre-mRNA (Fig. 5). After RRM deficiency, the RNA-binding ability of hnRNPA1 decreases, and the subcellular localization changes, thus reducing the ability to regulate the AS of *LOXL2* (Fig. 4). This is consistent with the *in vivo* splicing results of the *LOXL2* minigene by hnRNPA1-simulated phosphorylation of S91 and S95. Through the above *in vitro* and cell culture experiments, we conclude that the RRM1 and RRM2 domains have a significant impact on the splicing function of hnRNPA1. Deletion of either domain will affect the splicing of pre-mRNA by altering its subcellular localization and RNA-binding ability. The RRM domain in this way plays a key role in the AS of pre-mRNA through phosphorylation. The phosphorylation of S91 and S95 is closely related to the function of the RRM domains.

## Discussion

In this paper, we investigated the molecular mechanism of *LOXL2Δ13* production. As shown in the graphic abstract, there are two situations in the AS of *LOXL2* pre-mRNA. First, U1 and U2 snRNPs will bind to the 5′ SS and BP of exon 13 and catalyze the splicing of exon 13 to exon 14. This will result in the *LOXL2WT* isoform. Second, the S91/S95-unphosphorylated hnRNPA1 recognizes the UAG sequences of the 3′ SS and 5′ SS and binds to *LOXL2* exon 13, preventing proper splicing of exon 13 and ultimately excluding exon 13 from the mature mRNA along with the intron, resulting in the alternatively spliced *LOXL2Δ13* isoform. The binding of U1 and U2 snRNP to the 5′ SS and BPs is the beginning of the entire splicing process (36). If U1 and U2 are obstructed in binding to the splice site, this will affect the normal splicing of the pre-mRNA, resulting in alternatively spliced isoforms. Previous studies have found that hnRNPA1 can competitively bind to 5′ SSs, inhibiting the binding of U2AF2 (37). U2AF, composed of U2AF1 and U2AF2, is essential for the correct binding of U2 snRNP to the BP. Therefore, hnRNPA1 may inhibit the binding of U1 snRNP and U2AF at 5′ SSs and 3′ SSs, thereby inhibiting the splicing of *LOXL2* exon 13. This still requires further experimental verification.

Early studies on LOXL2 have shown that LOXL2 is a cancer-promoting factor that is highly expressed in a variety of tumors and can promote the proliferation, invasion, and metastasis of tumor cells (3). LOXL2Δ13 shows stronger cancer-promoting ability and also plays an important role in the treatment of obesity (6, 11). However, the expression of LOXL2Δ13 is much lower than that of LOXL2WT (Fig. 1*A*). Exon skipping involves differential associations of splicing factors and *cis*-acting elements, epigenetic modifications (N6-adenylate methylation (38), RNA editing (39), DNA methylation, and histone modification (40, 41)), and RNA structure (intron length and RNA stem-loop structure (42, 43, 44), pseudouridine modification (45), and GC content of genes (46, 47)). We speculated that *LOXL2Δ13* might occur as a result of alternative RNA splicing and that there might be something that causes *LOXL2Δ13* to be expressed at low levels. Previous studies on exon skipping mainly focus on *cis*-acting elements and splicing factors (48, 49, 50, 51). We sought the key splicing factors that regulate the AS of *LOXL2* and determined that hnRNPA1 regulates the AS of *LOXL2* exon 13.

HnRNPA1 can recognize and bind motifs containing UAG sequences (22, 30, 31). Among the 14 exons of *LOXL2* pre-mRNA, only the 3′ SS and 5′ SS of exon 13 have UAG sequences—UAGGUU and UAGGUA. Studies on splicing factorbinding motifs generally include the construction of minigene deletions or mutations (52, 53). The strength of splice sites also affects RNA splicing (52, 54). These two UAG sequences are located in the 3′ SS and 5′ SS, and when they are deleted, the strength of the splicing site is severely reduced, so they can only be mutated (Table S1). Therefore, minigene mutations must not change the strength of splice sites. We used ESEfinder 3.0 to predict the 5′ SS intensities after base mutation and finally selected the U13C+A14C mutation, which did not affect the 5′ SS intensities (Table S1). RNA-pulldown showed that a biotinylated probe including the U13C+A14C mutant bound very weakly to hnRNPA1, but the U13C+A14C mutation in the minigene only minimally changed minigene splicing (Figs. 3*I* and S3*C*). HnRNPA1 overexpression or knock down also did not significantly change the expression of *LOXL2WT* and *LOXL2Δ13*. We speculate that there might be two reasons. First, there might be several other splicing factors coregulating *LOXL2* splicing along with hnRNPA1. Second, the minigene only had mutations in the exon 13 5′ SS, where hnRNPA1 strongly bound but still had a WT 3′ SS. Thus, hnRNPA1 could still bind to the 3′ SS of exon 13 and regulate the splicing of *LOXL2*. The same gene-splicing event can also be regulated by multiple splicing factors. For example, splicing factors SRSF1, SRSF2, SRSF6, and PTBP1 can regulate the production of *BCL-XL* (53, 55, 56). In this study, hnRNPA1, SRSF9, SRSF10, and SRSF12 had significant regulatory effects on *LOXL2* minigene splicing. Therefore, there appears to be a complex network of splicing factors coregulating the splicing of *LOXL2*. This requires further research.

In terms of domains, RRM1 and RRM2 are reported to be domains where hnRNPA1 performs its splicing function, and both domains need to be present for RNA binding (33). Here, by RNA-pulldown and BLI, we demonstrate that hnRNPA1 requires the presence of both RRMs for strong RNA binding and AS of *LOXL2*. Phosphorylation of hnRNPA1 has been reported to affect the ability of RNA binding, subcellular localization, and telomere DNA replication (25, 26, 57). However, there are few reports on the effect of hnRNPA1 phosphorylation on splicing. Phosphorylation of hnRNPA1 on S91 and S95 results in the loss of hnRNPA1-mediated splicing due to cytoplasmic localization and reduced RNA-binding ability. Regarding the kinases upstream of hnRNPA1 S91 and S95, kinases phosphorylating S91 have not been reported, but S95 has been reported to be phosphorylated by PKC and DNA-PKcs (25, 57). However, the localization of these kinases in the cell is different, indicating that hnRNPA1 needs different kinases to perform different functions in different locations and different periods. Due to the large number of PKC protein isoforms, the large molecular weight of DNA-PKcs, and the numerous phosphorylation modification sites of hnRNPA1, we have not yet found the upstream kinase responsible for phosphorylating hnRNPA1 S91 and S95.

Recent studies have shown that hnRNPA1 enhances tubule formation and migration of lymphoendothelial cells through extracellular vesicle transport, thereby promoting metastasis of pancreatic cancer (58). *LOXL2WT* and *LOXL2Δ13* also promotes metastasis of tumor cells. Various hnRNP splicing factors are involved in the composition of stress granules and enhance the ability of cells to cope with stimuli (59). Whether hnRNPA1 can associate with RNPs that bind to *LOXL2* pre-mRNA and participate in the above process is still unknown. LOXL2 can promote tumor development and influence obesity by regulating glycometabolism and lipid metabolism (10, 11). HnRNPA1 can regulate the splicing of the key kinase *PKM* in glycolysis and participate in glycometabolism. All of these studies indicate that there is a strong association among hnRNPA1, *LOXL2WT*, and *LOXL2Δ13*. However, the process of RNA splicing is extremely complex, involves transcription, and also affects protein translation. The spliceosome is one of the most complex complexes in the cell. Multiple factors are involved, including the transcription rate of RNA polymerase II, the complex splicing factor regulatory network, and the influence of epigenetic modifications on the AS of *LOXL2* exon 13, all of which need further study. To sum up, we demonstrate that hnRNPA1 can regulate the AS of *LOXL2* and promote the expression of *LOXL2Δ13*.

## Experimental procedures

### Cell culture

Esophageal squamous cell carcinoma cell lines KYSE30, KYSE140, KYSE150, and KYSE510 (6, 60, 61) were cultured in RPMI 1640 medium (GIBCO, 31800-022) with 10% fetal bovine serum (FBS) (GIBCO, 10091-148). Human hepatoma cell lines HepG2 (SCSP-510) and HuH-7 (SCSP-526) were obtained from the National Collection of Authenticated Cell Cultures. HepG2 was cultured in Dulbecco’s modified Eagle’s medium (DMEM) (GIBCO, 12800-017) with 10% FBS, and HuH-7 was cultured in DMEM with 10% FBS, 1% Glutamax, 1% sodium pyruvate. The human non-small cell lung cancer cell line A549 and cervical cancer cell line HeLa were obtained from the National Collection of Authenticated Cell Cultures. A549 cells were cultured in RPMI 1640 medium with 10% FBS. HeLa and human embryonic kidney HEK293T cells (61) were cultured in DMEM with 10% FBS. All cells were incubated in a humidified atmosphere at 37 °C and 5% CO_2_. Cell lines were authenticated by short tandem repeat profiling and were routinely tested for *mycoplasma* contamination.

### Prediction of splicing factors and splice site strength

Predicted splicing factors near exon 13 of *LOXL2* pre-mRNA were obtained in ESEfinder 3.0 (https://esefinder.ahc.umn.edu/cgi-bin/tools/ESE3/esefinder.cgi) and SpliceAid (http://www.introni.it/splicing.html) databases. Prediction of splice site strength was obtained in ESEfinder 3.0. Detailed information on the input RNA sequence, predicted splicing factors, and splicing site strength can be found in Tables S2–S4.

### Plasmids

For construction of the *LOXL2* minigene, we used genomic DNA extracted from KYSE510 cells as the PCR template. The *LOXL2* minigene contained exons 12 to 14 and the intervening introns (https://www.ncbi.nlm.nih.gov/gene/4017). The *LOXL2* minigene was cloned into the pcDNA3 vector (62) at the *Eco*RI (Thermo Fisher Scientific, FD0274) and *XhoI* (Thermo Fisher Scientific, FD0694) restriction sites. The primers used were as follows. F: 5′-CAGTGTGCTGGAATTCACATCCAGAAGAATTACGAGTG-3′, R: 5′-TAGATGCATGCTCGAGCAAGTTTCAGTAAAAACCACAGG-3′. For the expression of splicing factors, we used complementary DNA (cDNA) extracted from HeLa cells as the cloning template for splicing factors SRSF1 to SRSF12, hnRNPA1 (hnRNPA1A), hnRNPA1B, and PTBP1. PCR products were cloned into the *Eco*RI and *Xho*I restriction sites of pcDNA3.1-N-SBP-HA (63) and pCMV-N-Flag (61). The site mutations of the *LOXL2* minigene and hnRNPA1 were based on PCR site-directed mutation. Domain deletions in hnRNPA1 were constructed by overlapping PCR. For the construction of hnRNPA1-His, RRM-His, RGG-His, S91A/S95A-His, and S91D/S95D-His plasmids, we used the previously constructed plasmids as templates. PCR products were cloned into the *Xho*I and *Bam*HI restriction sites of pET-32a-His (61). The cloned primer sequences are shown in Table S5. All DNA primers were purchased from BGI Genomics Co, Ltd, including the DNA primers below.

### *In vivo* splicing assay

In this study, minigenes were transfected three ways. According to the manufacturer's instructions, we used jetPRIME *in vitro* DNA & siRNA transfection reagent (Polyplus, 101000046) to transfect the minigenes. First, 0.8 μg WT or mutant minigene was transfected into HEK293T, HeLa, KYSE30, and other cells, then fresh culture medium was changed 6 to 18 h after transfection, and the total RNA extracted at 36 to 48 h. Second, 0.6 μg splicing factor and 0.4 μg minigene were cotransfected into cells, fresh culture medium was changed 6 to 18 h after transfection, and total cell protein and RNA were extracted 36 to 48 h after transfection. Third, 0.6 μg splicing factor, 50 nM siRNA, and 0.4 μg minigene were cotransfected, fresh culture medium was changed 6 to 18 h after transfection, and total cell protein and RNA were extracted 36 to 48 h after transfection. Western blot analysis of protein expression and RT-PCR analysis of the *LOXL2* minigene splicing were performed. The primers used were as follows. T7-F: 5′-TTAATACGACTCACTATAGGG-3′, *LOXL2*-minigene-test-R-2: 5′-GACAGCTGGTTGTTTAAGAG-3′.

### RNAi and transfection

According to the manufacturer's instructions, 50 nM siRNA was transfected into KYSE30, KYSE150, and A549 cells using jetPRIME *in vitro* DNA & siRNA transfection reagent. Culture medium was changed 12 h after transfection, and total cell protein or RNA was extracted 48 h after transfection. The sense siRNA sequences of human hnRNPA1 were siHNRNPA1#1: 5′-CAGCUGAGGAAGCUCUUCATT-3′, siHNRNPA1#4: 5′-AGAUAUUUGUUGGUGGCAUUATT-3′. All RNA (siRNA and single-stranded RNA oligos) were purchased from Suzhou GenePharma Co, Ltd

### Western blotting

1× Laemmli sample buffer (Bio-Rad, 1610747) was used to lyse cells and extract total protein. Protein samples were loaded onto a 10% polyacrylamide gel and then transferred to a polyvinylidene difluoride (PVDF) membrane (Millipore). The PVDF membrane was blocked in 5% skimmed milk (prepared in Tris-buffered saline and Tween-20, abbreviated as TBST, which contains 20 mM Tris, 137 mM NaCl, 0.1% Tween-20, pH 7.6) for 1 h, followed by the addition of primary antibody for 12 h at 4 °C. The next day, the membrane was washed with TBST and incubated with horseradish peroxidase-conjugated secondary antibody at room temperature for 2 h. Antigen-antibody complexes were detected with a StarSignal Chemiluminescent Assay Kit (GenStar, E171). Gray-scale values of proteins on the PVDF membrane were measured by ImageJ software (https://wsr.imagej.net/distros/win/ij154-win-java8.zip). The following antibodies were used in this study: E-1 for lamin A/C (Santa Cruz, sc-376248), anti-HA (TransGen Biotech, HT301), anti-DYKDDDDK (TransGen Biotech, HT201), anti-His (TransGen Biotech, HT501), anti-GAPDH (Proteintech, 60004-1-Ig), anti-beta actin (Proteintech, 60008-1-Ig), D21H11 for hnRNPA1 (Cell Signaling Technology, 8443), and 9H10 for hnRNPA1 (Abcam, ab5832).

### RT-PCR and RT-qPCR

According to the manufacturer's instructions, total RNA was extracted from cells with TRIzol (Invitrogen, 15596026), and reverse transcription was performed with a HiScript III 1st Strand cDNA Synthesis Kit (Vazyme, R312). For RT-PCR, 2 × Taq PCR StarMix (GenStar, A012) was used to amplify the expressed alternatively spliced forms of *LOXL2*. The PCR product was subjected to agarose gel electrophoresis and the images were captured by a ChemiDoc Touch Imaging System (Bio-Rad). The gray-scale values of *LOXL2WT* and *LOXL2Δ13* on the agarose gel were measured by ImageJ software for quantification. For RT-qPCR, ChamQ Universal SYBR qPCR Master Mix (Vazyme, Q711) and ABI 7500 (Applied Biosystems) were used to detect the expression of target genes. The 2^−ΔΔCt^ method was used to analyze the data and quantify gene expression. The primers used are shown in Table S6.

### RNA immunoprecipitation

In order to immunoprecipitate the endogenous ribonucleoprotein complex from whole cell lysate, RIP buffer was used to lyse KYSE140 cells. The components of RIP buffer were 50.0 mM Tris–HCl pH7.5, 150.0 mM NaCl, 5.0 mM EDTA, 0.5% vol/vol NP-40, and 1.0% vol/vol Triton X-100, and an appropriate amount of RNase inhibitor (New England Biolabs, M0307) and protease inhibitor (MedChemExpress, HY-K0010) were added. Anti-hnRNPA1 antibody or IgG (Cell Signaling Technology, 2729) were incubated overnight at 4 °C with whole cell lysate, then 50 μl Protein A/G magnetic beads (MedChemExpress, HY-K0202) was added to the antibody-protein mixture, and then incubated at 4 °C for 4 h. After washing the magnetic beads three times with RIP buffer, 1 ml Trizol was added to the magnetic beads, and RNA was extracted according to the manufacturer's instructions (Invitrogen, 15596026). Finally, RT-PCR analysis was performed. HA-hnRNPA1 was transfected into KYSE150 cells before immunoprecipitation of exogenous ribonucleoprotein complexes. Anti-HA magnetic beads (MedChemExpress, HY-K0201) were used to immunoprecipitate HA-hnRNPA1. The detection primers used were LOXL2-RIP-test-F: 5′-TGGAGGTGGCCGCAGAGGGAACAAGCA-3′, LOXL2-RIP-test-R: 5′- TTAGGAAGCTGCCTCTGGGTCCTTGAGAAA-3′.

### RNA-pulldown assay

Whole cell lysates of HeLa, KYSE150, and KYSE510 cells were prepared using RNA-pulldown lysates. The components of the RNA-pull down lysate buffer were 150.0 mM KCl, 0.5 mM DTT, 0.5% vol/vol NP-40, and 25.0 mM Tris–HCl, pH 7.5, and an appropriate amount of RNase inhibitor and protease inhibitor were added. Then, 0.4 μM biotin-labeled RNA oligonucleotides were bound to 25 μl streptavidin magnetic beads (MedChemExpress, HY-K0208). The binding reaction was carried out in binding buffer I (10.0 mM Tris–HCl pH7.5, 1.0 mM EDTA, 1.0 M NaCl, 0.01–0.1% Tween-20) with an appropriate amount of RNase inhibitor and protease inhibitor added. After the RNA-magnetic bead mixture was rotated and incubated at 4 °C for 2 h, the magnetic beads were washed 3 times with buffer I. Then, the magnetic bead–RNA complex was mixed with whole cell lysate and then incubated overnight at 4 °C. The following morning, 1 × Laemmli sample buffer was added to elute the RNA-binding protein for Western blot analysis. The biotin-labeled RNA oligonucleotides used were as follows. Oligo 1: AGCAGCCUCCUCUGCCCUGCUUCUCCCUAGGUUGUU, Oligo 2: ACAACUGCCACAUAGGUAAGGCCAGGCCGC, and Oligo 2-U13C+A14C (U13C+A14C): ACAACUGCCACACCGGUAAGGCCAGGCCGC.

### Protein purification and quantification

His-tagged protein purification and quantification was performed as described below. We transformed plasmids into Transetta (DE3) Chemically Competent Cell (TransGen Biotech, CD801-03) and picked a clone. The positive clone was cultured in LB medium (10 g/L tryptone, 5 g/L yeast extract, 10 g/L NaCl) at 37 °C until an OD_600_ nm value of 0.5 to 1.0 was reached. Then, cells were induced with 0.5 mM IPTG (Amresco, 0487) at 16 °C for 16 h. After centrifugation 5000 rpm for 5 min, the supernatant was discarded, and bacteria were lysed on ice for 30 min in His lysis buffer (50 mM sodium phosphate (pH 8.0), 300 mM NaCl, 10 mM imidazole) and 1 mg/ml lysozyme (Sigma, 62971). Ultrasonically broken bacteria were centrifuged at 10,000 rpm for 10 min, and the supernatant was collected and incubated with His Bind Resin (Millipore, 70666-3) at 4 °C for 3 h. Protein-bound resin was centrifuged at 4 °C, 1000 rpm, and the supernatant was discarded. Resin was then washed five times with His buffer. Finally, the resin was eluted with His eluting buffer (50 mM sodium phosphate (pH 8.0), 300 mM NaCl, and 200 mM imidazole) at 4 °C for 1 h. The eluate was centrifuged at 1000 rpm at 4 °C, and the supernatant containing purified protein was collected. All purified proteins were quantified using a Pierce BCA Protein Assay Kit (Thermo Fisher Scientific, 23225) according to the protocol provided by the manufacturer.

### Immunofluorescence

HA-hnRNPA1 was transfected into HeLa cells. After 48 h, cells were washed with PBS (137 mM NaCl, 2.7 mM KCl, 4.3 mM Na_2_HPO_4_, 1.4 mM KH_2_PO_4_, pH7.2∼7.4), then fixed with 0.4% paraformaldehyde at 4 °C for 10 min. To permeabilize the cells, cells were incubated in 0.1% Triton X-100 at 4 °C for 10 min. Then, 5% bovine serum albumin (Sangon Biotech, A500023-0100) was used to block nonspecific binding at room temperature for 1 h, then cells were incubated with anti-HA antibody (TransGen Biotech, HT301, 1:500 dilution) at 4 °C overnight. The next day, cells were incubated with Alexa Fluor 488-conjugated donkey anti-mouse IgG (H+L) secondary antibody (Invitrogen, A-21202, 1:800 dilution) at room temperature in the dark for 1 h. Subsequently, DAPI (Santa Cruz, sc2084, 1:1500 dilution) was used for counter-staining at room temperature in the dark for 5 min, then images were taken using a laser confocal microscope (Zeiss, LSM800).

### Nuclear and cytoplasmic extraction

Cytoplasmic components of KYSE30 and HeLa cells were extracted using hypotonic buffer A (10 mM Tris–HCl pH7.5, 2 mM MgCl_2_, 3 mM CaCl_2_, 320 mM sucrose, 1 mM DTT, 0.3% NP40). Cells were washed once with pre-cooled PBS, and then pre-cooled buffer A containing protease inhibitor was added. The cells were scraped off the dish with a cell scraper, lysed at 4 °C for 10 min, and pelleted by centrifugation (4 °C, 2800*g*, 5 min). The supernatant was collected in a 1.5 ml centrifuge tube and served as the cytoplasmic component. The pellet obtained by centrifugation was the crude nuclear extract. The pellet was washed three times by adding pre-cooled buffer A containing protease inhibitors and centrifuging at 4 °C for 5 min at 2800*g*. After the last centrifugation, the supernatant was discarded to obtain the nuclear extract. After cell fractionation, Western blotting was performed.

### BLI assay

The expression and purification of hnRNPA1-His, S91A/S95A-His, S91D/S95D-His, ΔRRM1-His, ΔRRM2-His, and ΔRGG-His were described previously. The kinetic characterization was conducted in interaction buffer (PBS with 0.002% Tween-20 and 0.02% bovine serum albumin, dissolved and filtered). This experiment was implemented in two ways. First, biotin-oligo1, -oligo2, and -U13C+A14C were diluted to 1 μM in interaction buffer. The purified proteins were diluted to 30 μg/ml, 10 μg/ml, 3 μg/ml, 1 μg/ml, and 0.3 μg/ml in interaction buffer. The streptavidin probes were hydrated in interaction buffer for 10 min before use. Following an initial baseline of 60 s, the streptavidin probes were loaded with biotin-RNAs for 80 s. Then, the probes were immersed in solution containing His-proteins for 120 s, to measure the association between biotin-RNAs and His-proteins, and then transferred to interaction buffer for dissociation for 120 s. One of the probes recorded a buffer reference signal for background subtraction. Second, the His-proteins were diluted to 30 μg/ml in interaction buffer, and the biotin-RNAs were diluted to 300 nM in interaction buffer. The Ni-NTA probes were hydrated in interaction buffer for 10 min before use. Following an initial baseline of 60 s, the Ni-NTA probes were loaded with His-proteins for 80 s. Then, the probes were immersed in solution containing the biotin-RNAs for 54 s to measure the association between His-proteins and biotin-RNAs and then transferred to interaction buffer for dissociation for 120 s. One of the probes recorded a buffer reference signal for background subtraction. The results were analyzed with Gator Part11 software (21 CFR Part 11 Compliant BLI software, https://www.gatorbio.com/products/software/).

### Proteomics and phosphoproteomic data analysis

The proteomic data of 124 patients with esophageal cancer and label-free phosphoproteomics data of 31 pairs of tumor and nontumor esophageal tissues were derived from data published by our laboratory in 2021 (29). Raw files of the proteome and phosphoproteome datasets can be obtained from the PRIDE database (accession number PXD021701) or iProX database (accession number IPX0002501000) (64, 65). In this paper, these proteomic data were only used to analyze the expression and phosphorylation of hnRNPA1.

### Statistical analysis

Data were statistically analyzed using ImageJ and GraphPad Prism 8.0 (GraphPad Software). The mean ± SD of the data of each group was obtained. The *t* test was used to test the difference between the data of each group (with *p* < 0.05 indicating statistical significance).

## Data availability

The data presented in this study are available in the article.

## Supporting information

This article contains supporting information.

## Conflict of interest

The authors declare that they have no conflicts of interest with the contents of this article.
