# Supplementary material for: Splicing factor hnRNPA1 regulates alternative splicing of LOXL2 to enhance the production of LOXL2Δ13
Source: J Biol Chem. 2024 May 27;300(7):107414. doi: 10.1016/j.jbc.2024.107414 (PMC11259713; doi:10.1016/j.jbc.2024.107414)
Supplement: Supporting Information [file mmc2.docx]

**SUPPORTING INFORMATION**

**Splicing factor hnRNPA1 regulates alternative splicing of *LOXL2* to enhance production of *LOXL2Δ13***

**Deyuan Pan**^1,2,3,‡^**, Lin Long**^1,2,3,‡^**, Chengyu Li**^1,3^**, Yingxin Zhou**^1,3^**, Qing Liu**^2^**, Ziting Zhao**^1^**, Hui Zhao**^1,3^**, Wan Lin**^3^**, Zhenyuan Zheng**^1,3^**, Liu Peng**^1,3^**, Enmin Li**^1,3,*^ **and Liyan Xu**^1,3,4,*^

*From the* ^1^*Key Laboratory of Molecular Biology for High Cancer Incidence Coastal Chaoshan Area, Department of Biochemistry and Molecular Biology, Shantou University Medical College, Shantou, Guangdong Province, China;* ^2^*State Key Laboratory of Pathogenesis, Prevention and Treatment of High Incidence Diseases in Central Asia, Xinjiang Medical University, Urumqi, China;* ^3^*Institute of Basic Medical Science, Cancer Research Center, Shantou University Medical College, Shantou, Guangdong Province, China;* ^4^*Institute of Oncologic Pathology, Shantou University Medical College, Shantou, Guangdong Province, China*

^‡^ These authors contributed equally to this work.

* For correspondence: Liyan Xu, [lyxu@stu.edu.cn](mailto:lyxu@stu.edu.cn); Enmin Li, nmli@stu.edu.cn.

**content**

[**Supplementary Figures**](#_Toc165407041)

[**Figure S1. Construction of the *LOXL2* minigene and prediction of splicing factors**](#_Toc165407042)

[**Figure S2. In vivo splicing of the *LOXL2* minigene in HeLa, A549 and KYSE150 cells**](#_Toc165407043)

[**Figure S3. 5′ SS and 3′ SS of *LOXL2***](#_Toc165407044)

[**Figure S4. *HNRNPA1*, *SRSF9*, *SRSF10*, *SRSF12V1*, *LOXL2WT* and *LOXL2Δ13* mRNA expression**](#_Toc165407045)

[**Figure S5. hnRNPA1 without RRM is unable to bind RNA**](#_Toc165407046)

[**Figure S6. Subcellular localization of domain-deleted hnRNPA1s**](#_Toc165407047)

[**Figure S7. Differential phosphorylation of hnRNPA1 in esophageal carcinoma**](#_Toc165407048)

[**Figure S8. The hnRNPA1 (S91D/S95D) mutant is unable to regulate LOXL2 exon13 splicing**](#_Toc165407049)

[**Figure S9. Phosphorylation of S91 and S95 affects the RNA binding capacity of hnRNPA1**](#_Toc165407050)

[**Figure S10. Phosphorylation of S91 and S95 affects subcellular localization of hnRNPA1**](#_Toc165407051)

[**Supplementary Tables**](#_Toc165407052)

[**Supplementary Table S1. UAG sequence mutations in LOXL2 exon 13**](#_Toc165407053)

[**Supplementary Table S5. Plasmids and gene cloning primers used in this paper**](#_Toc165407054)

[**Supplementary Table S6. Additional primers**](#_Toc165407055)

[**Supplementary Table S7. The siRNAs used in this paper**](#_Toc165407056)

[**Supplementary Table S8. Biotinylated RNAs used in this paper**](#_Toc165407057)

# Supplementary Figures

**
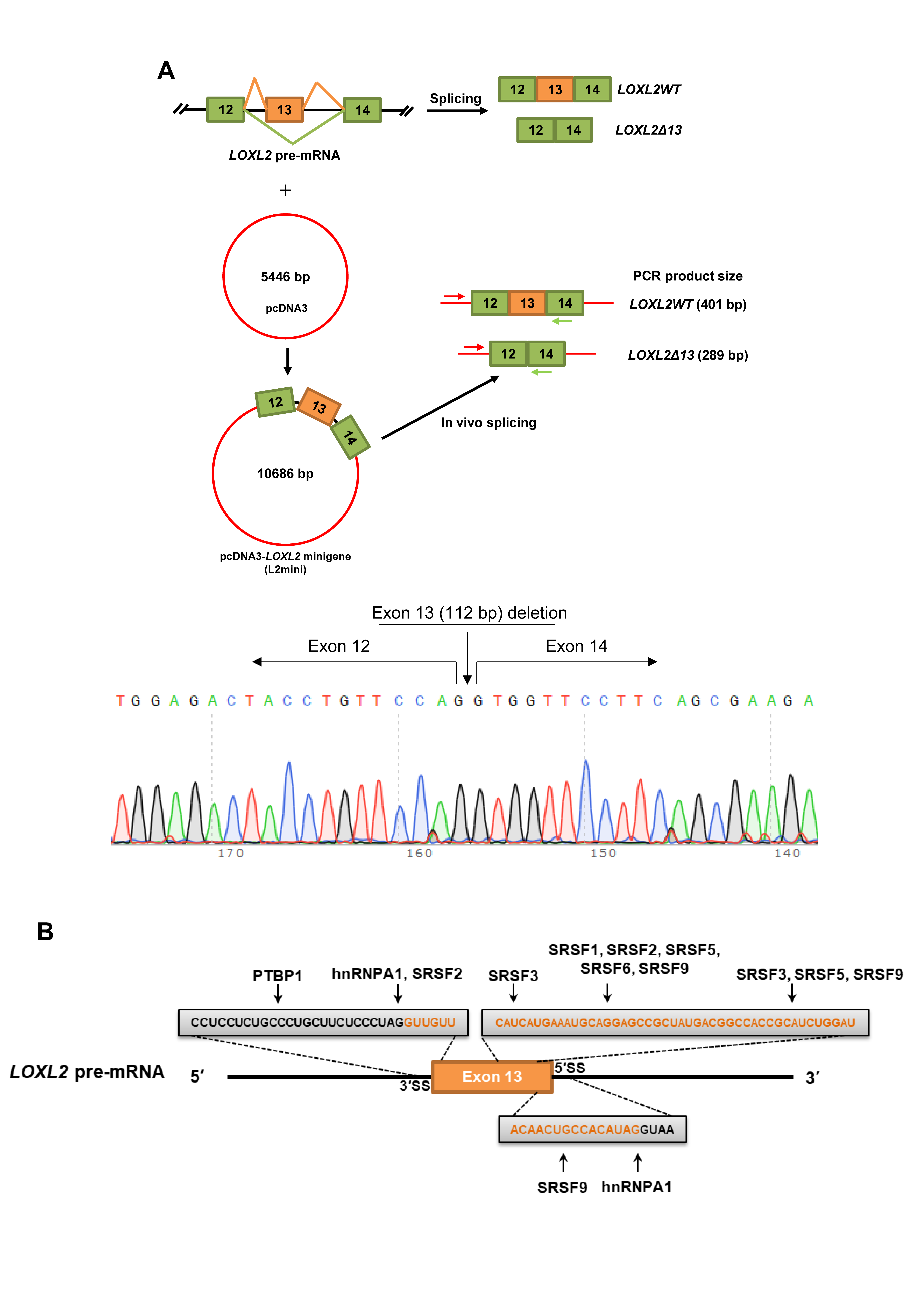
**

Figure S1. Construction of the *LOXL2* minigene and prediction of splicing factors**.** (**A**) The above schematic diagram shows the alternative splicing pattern of human *LOXL2* exon 13 and construction of the *LOXL2* minigene. The following schematic diagram shows the sequencing results of two splicing isoforms formed by the *LOXL2* minigene in cells. Boxes represent exons, and black lines between the boxes represent introns. The orange and green lines above the intron indicate the pattern of RNA splicing. The red arrow indicates the forward primer, which was located on the carrier, and the green arrow indicates the reverse primer, which was located at the 5′ end of exon 14. (**B**) ESEfinder 3.0 (https://esefinder.ahc.umn.edu/cgi-bin/tools/ESE3/esefinder.cgi)- and SpliceAid (http://www.introni.it/splicing.html)-predicted splicing factors binding near exon 13 of *LOXL2* pre-mRNA. Dashed arrows indicate the binding position of the splicing factors.


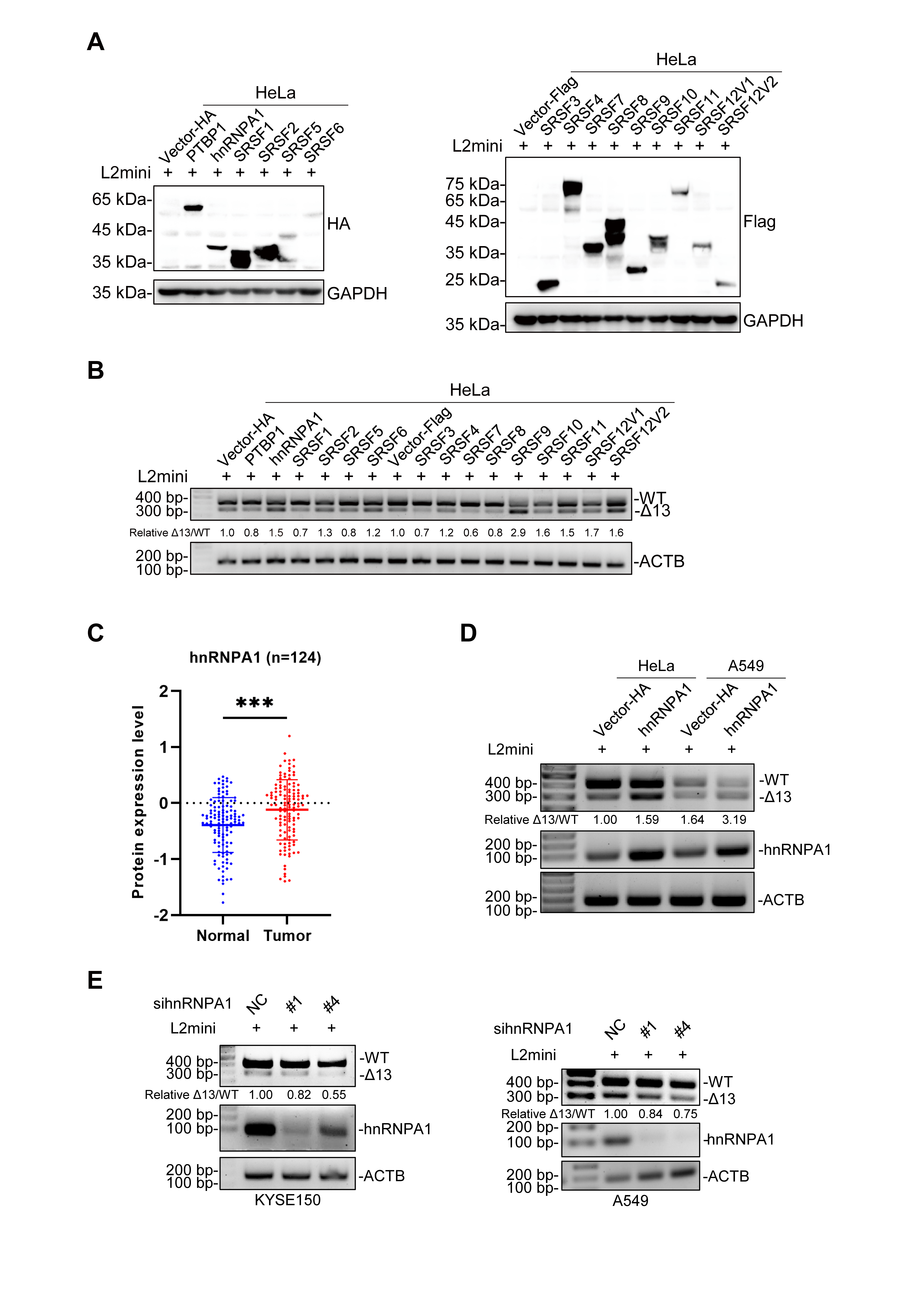


Figure S2. In vivo splicing of the *LOXL2* minigene in HeLa, A549 and KYSE150 cells**.** (**A**) Western blot showing the overexpression of splicing factors SRs 1-12, PTBP1 and hnRNPA1 in HeLa cells. (**B**) Splicing factors co-transfected with the *LOXL2* minigene in HeLa cells. Relative Δ13/WT represents the ratio of the gray-scale values of the bands for *LOXL2Δ13* and *LOXL2WT*. *ACTB* was used as an internal control for sample loading. (**C**) Protein expression levels of hnRNPA1 in normal and tumor tissues in 125 patients with esophageal cancer. ****P* < 0.001. (**D**) Overexpression of hnRNPA1 in HeLa and A549 cells regulated *LOXL2* minigene splicing. HnRNPA1 was characterized by RT-PCR. (**E**) Effects of knockdown of endogenous hnRNPA1, in KYSE150 and A549 cells, on *LOXL2* minigene splicing. Relative Δ13/WT represents the ratio of band gray values for *LOXL2Δ13* and *LOXL2WT*. *ACTB* was used as an internal control for sample loading.

**
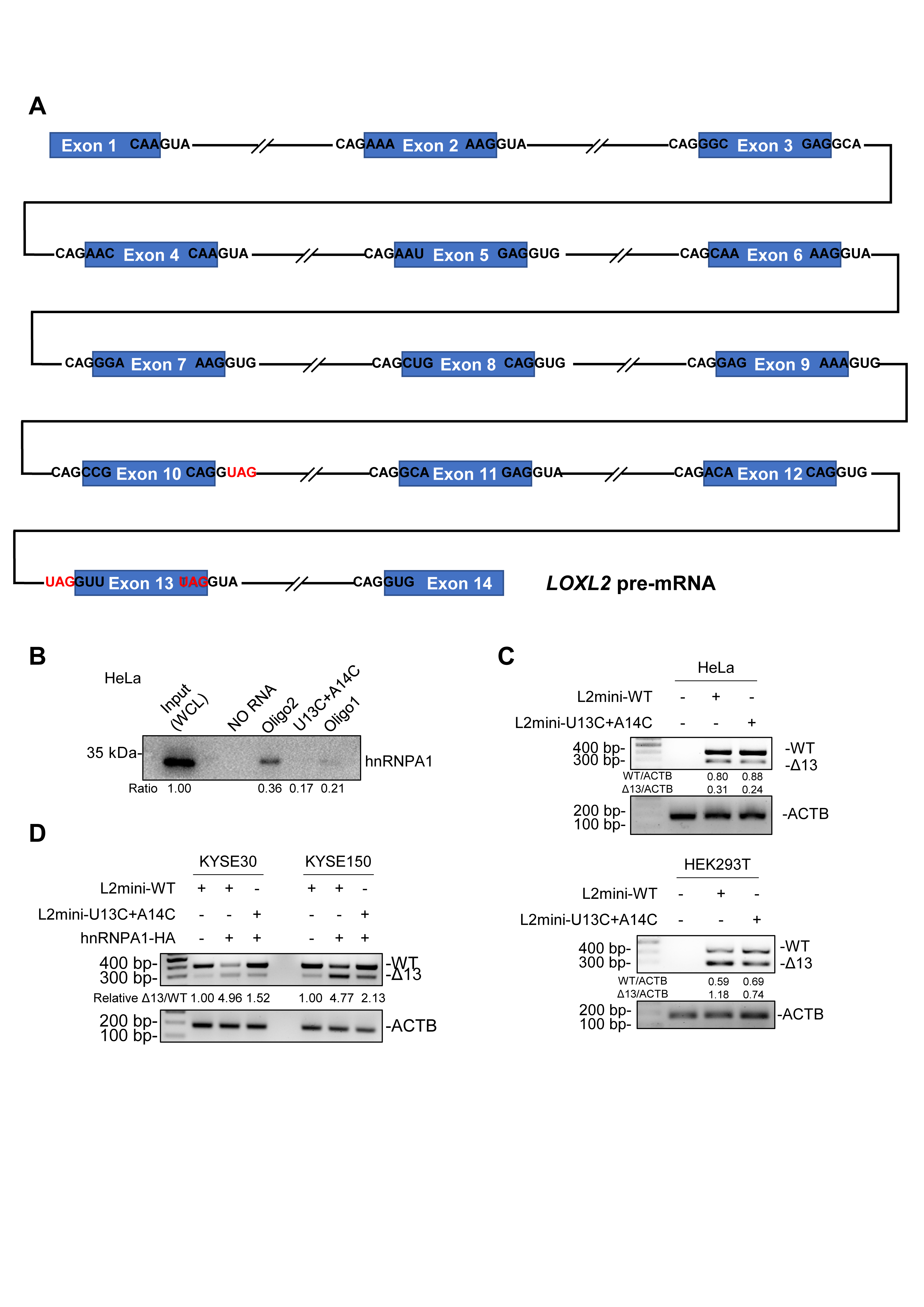
**

Figure S3. 5′ SS and 3′ SS of *LOXL2***.** (**A**) *LOXL2* contains 14 exons. The nucleotide sequences near the 5′ and 3′ ends of each exon are shown. Red indicates UAG sequences. (**B**) RNA-pull down was carried out in HeLa cells to verify the binding site of hnRNPA1. Ratio refers to pulldown/input, with input as the benchmark. (**C**) Splicing of the vector (pcDNA3), and wild-type and U13C+A14C mutant *LOXL2* minigenes in HeLa and HEK293T cells. WT/ACTB represents the gray scale ratio of *LOXL2WT* and ACTB, and Δ13/ACTB represents the gray scale ratio of *LOXL2Δ13* and ACTB. ACTB was used as the internal control for sample loading. (**D**) Effects of overexpression of hnRNPA1 on the splicing of the wild-type and U13C+A14C mutant *LOXL2* minigenes in KYSE30 and KYSE150 cells, Relative Δ13/WT represents the ratio of the gray-scale values of the bands for *LOXL2Δ13* and *LOXL2WT*. *ACTB* was used as an internal control for sample loading.

**
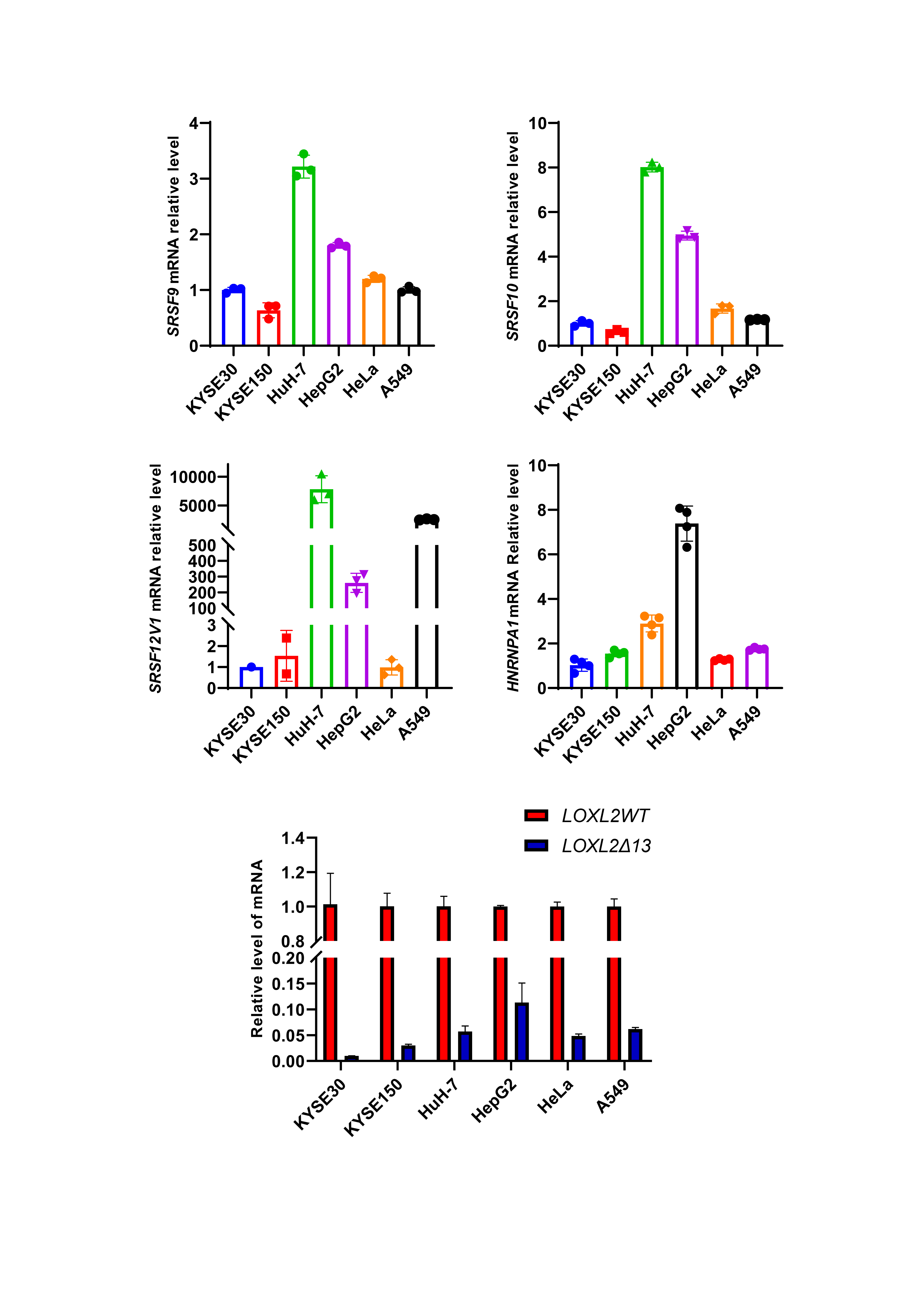
**

Figure S4. *HNRNPA1*, *SRSF9*, *SRSF10*, *SRSF12V1*, *LOXL2WT* and *LOXL2Δ13* mRNA expression**.** By qPCR, we detected the expression of *HNRNPA1*, *SRSF9*, *SRSF10*, *SRSF12V1*, *LOXL2WT* and *LOXL2Δ13* in KYSE30, KYSE150, HuH-7, HeLa and A549 cells. The results of each group are expressed as mean ± SD.

**
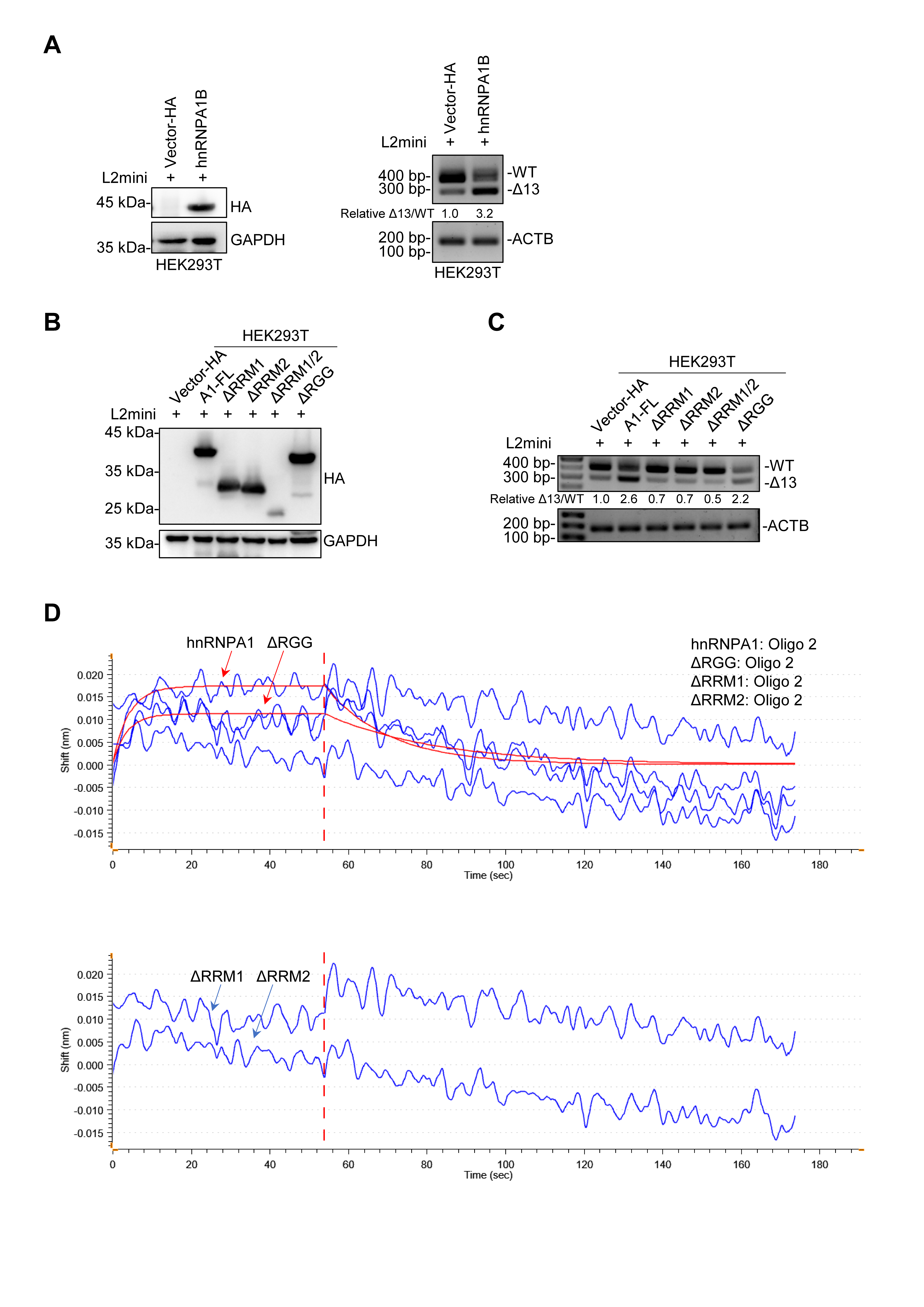
**

Figure S5. hnRNPA1 without RRM is unable to bind RNA**.** (**A**) Effect of hnRNPA1B on splicing of the *LOXL2*-minigene. (**B**) Expression of hnRNPA1 full-length and deletion mutants in HEK293T. pcDNA3.1-N-SBP-HA and pCMV-N-Flag as vectors. (**C**) RT-PCR was used to determine the effect of different deleted hnRNPA1 domains on hnRNPA1-mediated splicing. Relative Δ13/WT ratios were calculated. (**D**) Biolayer interferometry was used to calculate the kinetic characterization of hnRNPA1-His, ΔRRM1-His, ΔRRM2-His, and ΔRGG-His with oligo2. Ni-NTA probes were used to bind His-proteins. HnRNPA1 without either RRM cannot fit the binding curve. The images were recorded by biolayer interferometry software. The blue curve represents the real-time combination curve, and the red curve represents the fitted combination curve. The dashed line represents a time of 54 seconds. The above image shows the binding of hnRNPA1-His, ΔRRM1-His, ΔRRM2-His, and ΔRGG-His to oligo2, while the following image shows the binding of ΔRRM1-His and ΔRRM2-His to oligo2.

**
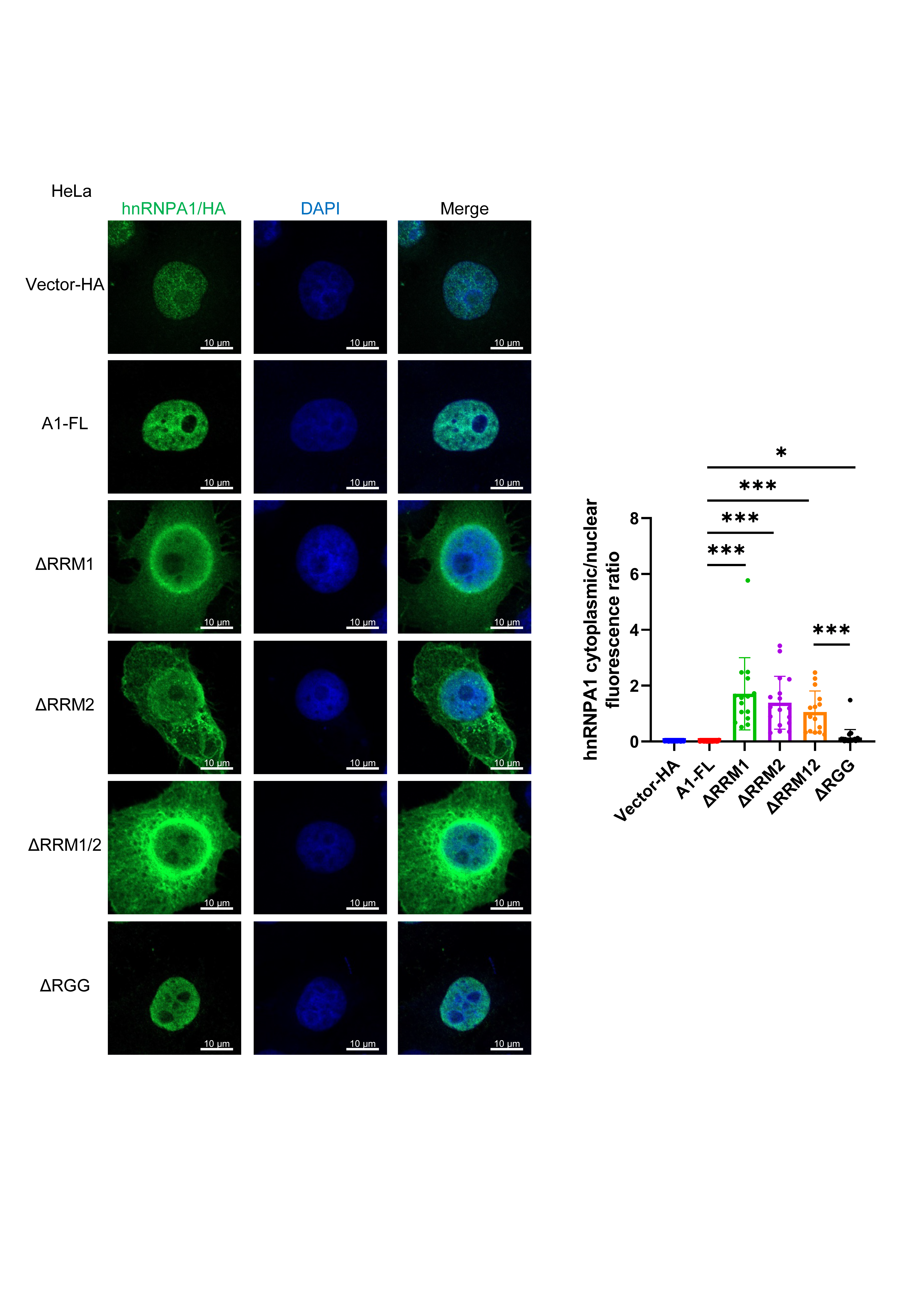
**

Figure S6. Subcellular localization of domain-deleted hnRNPA1s**.** The subcellular localization of domain-deleted hnRNPA1s was verified by immunofluorescence. The image on the left shows typical plots of the distribution of hnRNPA1 in each group of cells. The graph on the right is a statistical graph. Each group counted consisted of 10-25 cells and the plasma to nucleus ratio of hnRNPA1 in each cell was calculated using ImageJ. Green indicates hnRNPA1, blue DAPI shows the nucleus. The control group was stained with hnRNPA1 antibody. The results of each group are expressed as mean ± SD. **P* < 0.05, ***P* < 0.01, ****P* < 0.001.

**
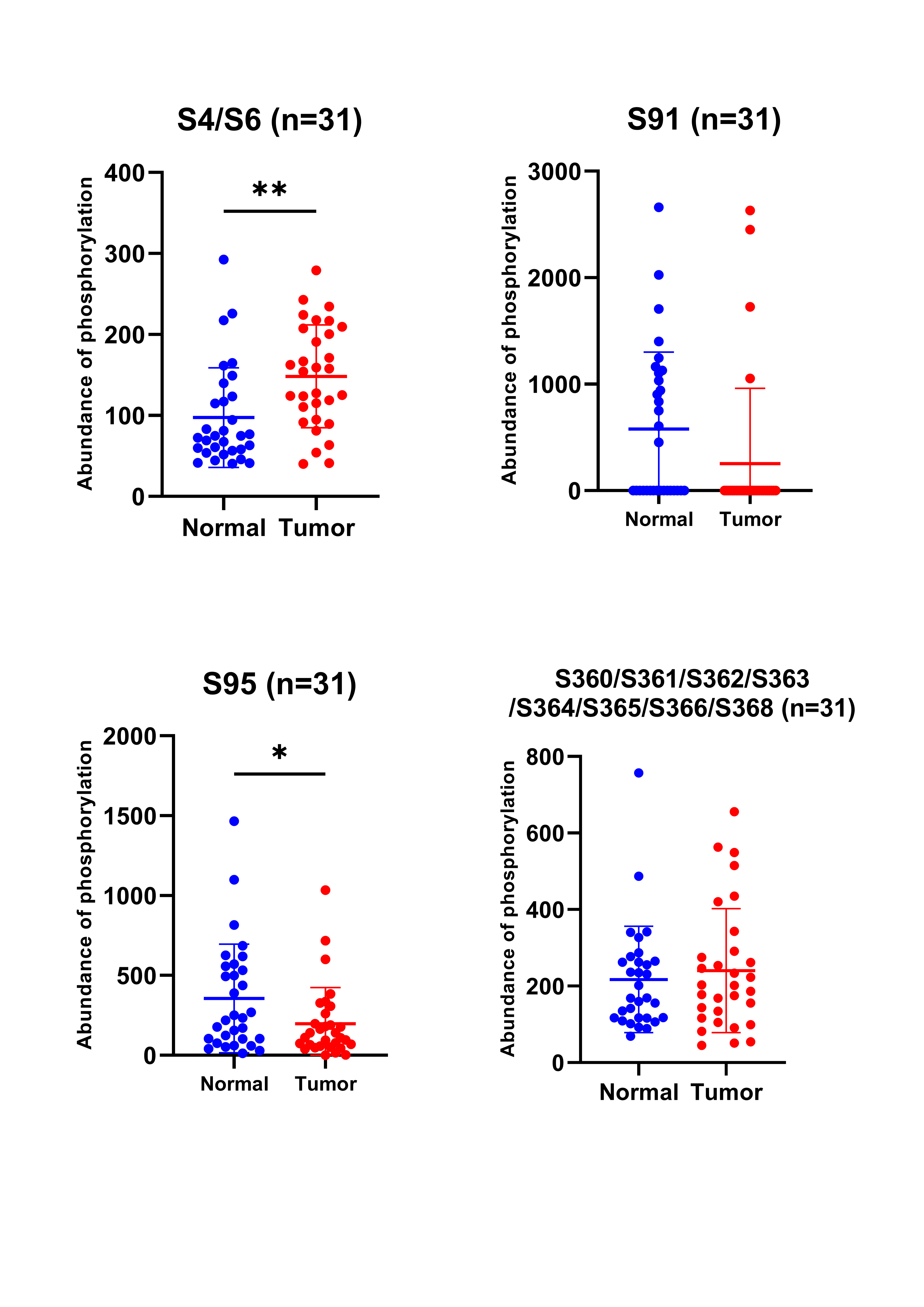
**

Figure S7. Differential phosphorylation of hnRNPA1 in esophageal carcinoma**.** Differential phosphorylation of hnRNPA1 in phosphoproteomics profiling from 31 cases of esophageal cancer. The figure shows the abundance of phosphorylation at hnRNPA1 in normal and tumor tissues. **P* < 0.05, ***P* < 0.01.

**
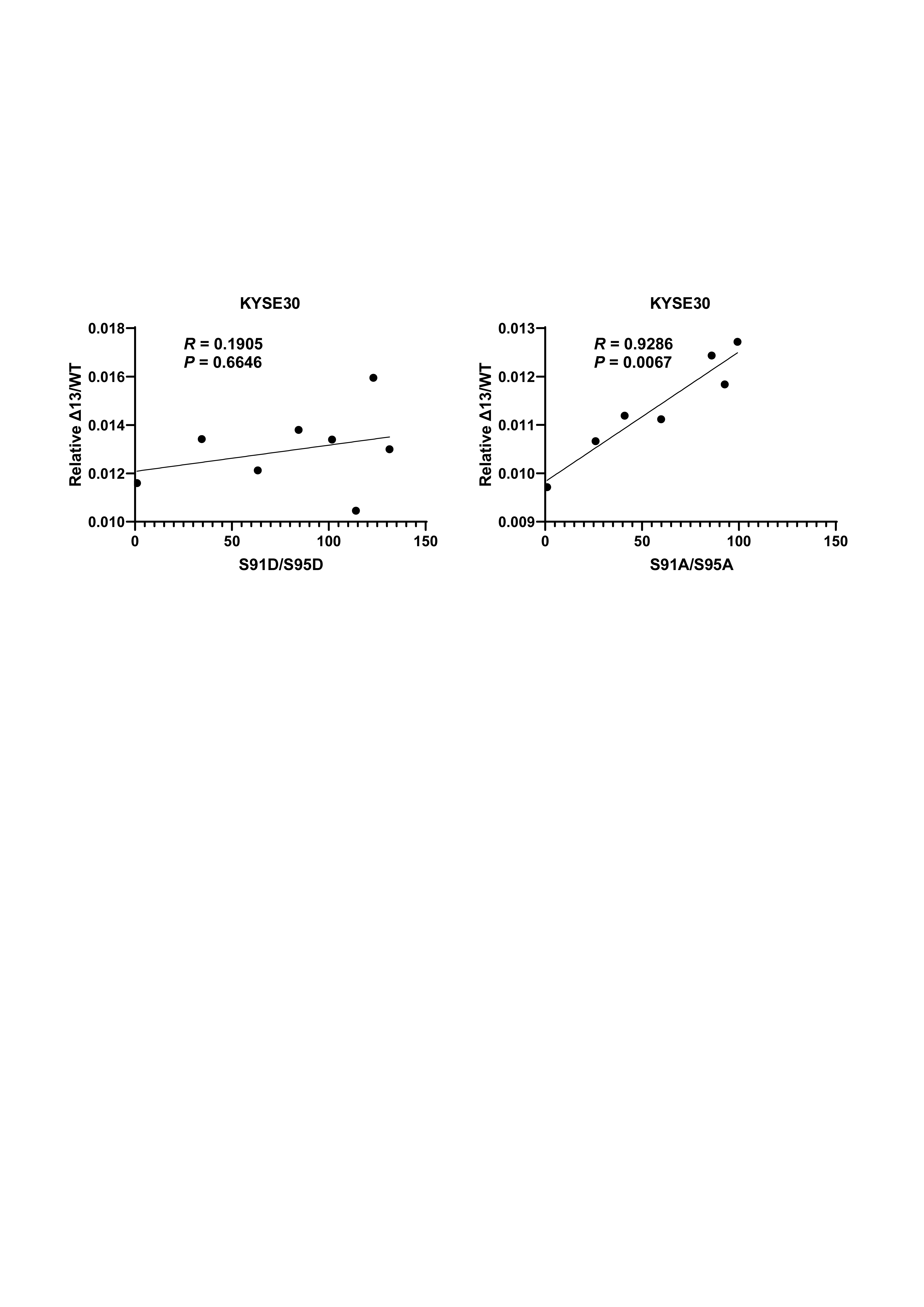
**

Figure S8. The hnRNPA1 (S91D/S95D) mutant is unable to regulate LOXL2 exon13 splicing**.** RT-qPCR was used to detect the RNA levels of hnRNPA1 (S91A/S95A) and (S91D/S95D) mutants, *LOXL2WT* and *LOXL2Δ13* in KYSE30 and KYSE150 cells. S91A/S95A- and S91D/S95D-mutant hnRNPA1s were transfected into cells (transfected plasmid amounts: 0.6, 0.8, 1.0, 1.2, 1.4, 1.6, 1.8 μg). Spearman correlation was used to analyze the correlation between mutant hnRNPA1 and endogenous relative Δ13/WT.

**
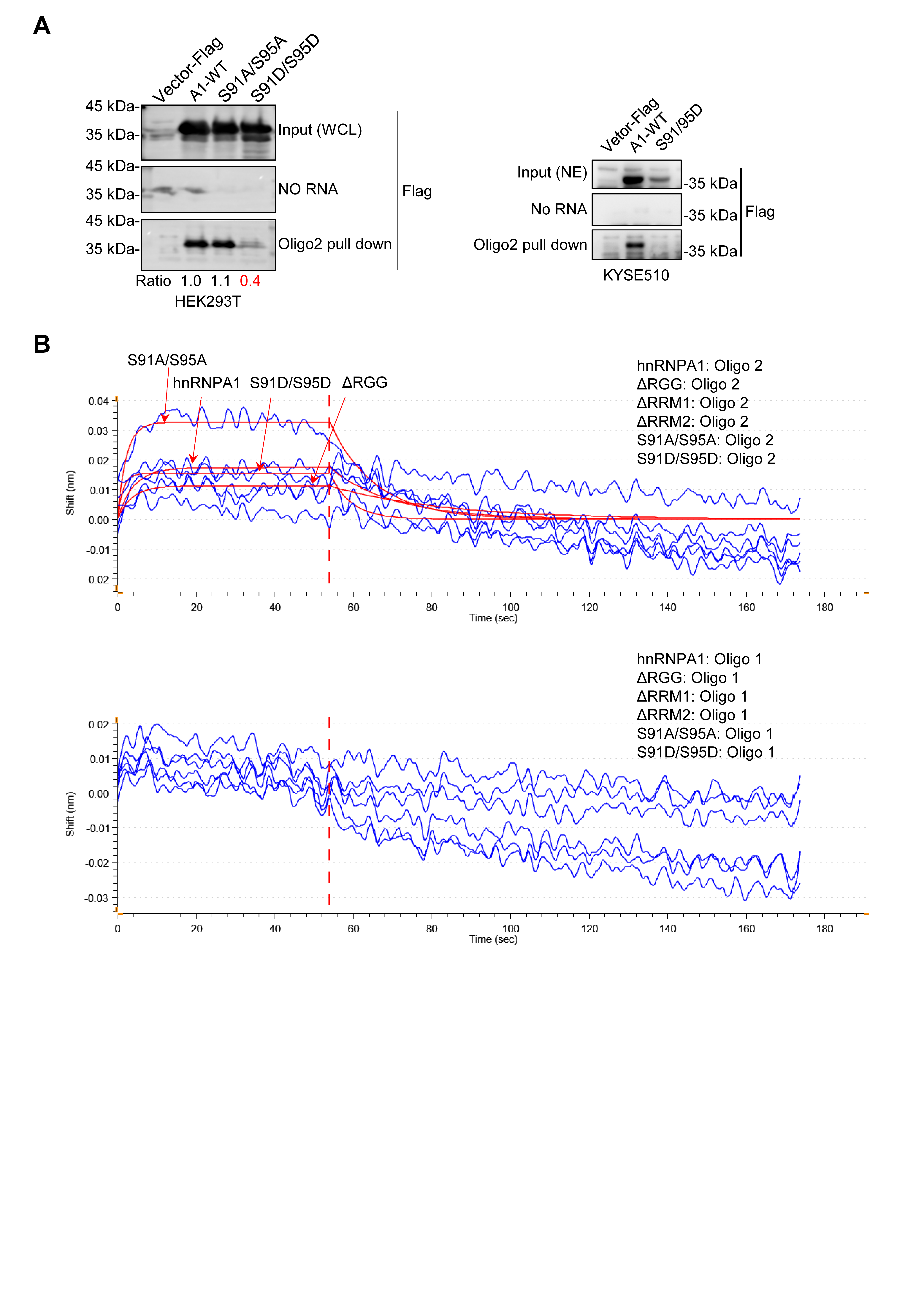
**

Figure S9. Phosphorylation of S91 and S95 affects the RNA binding capacity of hnRNPA1**.** (**A**) RNA-pulldown was carried out in HEK293T and KYSE150 cells to determine the binding of simulated phosphorylation and simulated dephosphorylation of hnRNPA1 to RNA. Ratio refers to pulldown/input. (**B**) Biolayer interferometry was used to calculate the kinetic characterization of hnRNPA1-His, ΔRRM1-His, ΔRRM2-His, ΔRGG-His, S91A/S95A-His and S91D/S95D-His with oligo1 and oligo2. Ni-NTA probes were used to bind His-proteins. HnRNPA1 without RRM cannot fit the binding curve. The images were recorded by biolayer interferometry software. The blue curve represents the real-time combination curve, and the red curve represents the fitted combination curve. The dashed line represents a time of 54 seconds. The above image shows the binding of His-proteins to oligo2, while the following image shows the binding of His-proteins to oligo1.

**
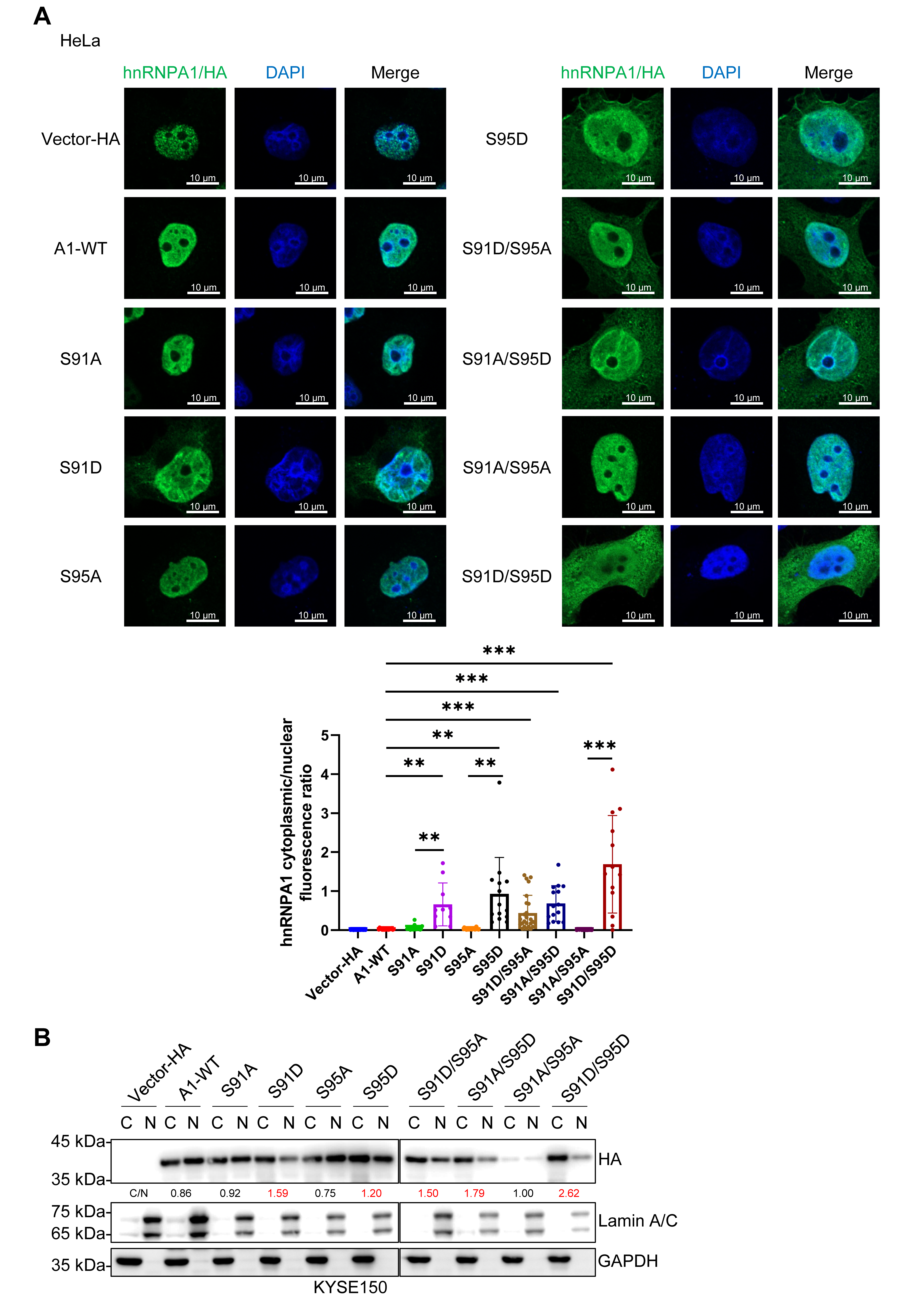
**

Figure S10. Phosphorylation of S91 and S95 affects subcellular localization of hnRNPA1**.** (**A**) Subcellular localization of hnRNPA1 phospho- and non-phosphorylatable mimics at S91 and S95 shown by immunofluorescence. The image on the left shows typical plots of the distribution of hnRNPA1 in each group of cells. The graph on the right is a statistical graph. Each group counted was comprised of 10-25 cells and calculated the plasma to nucleus ratio of hnRNPA1 in each cell using ImageJ. Green indicates hnRNPA1, blue DAPI shows the nucleus. The control group was labeled with endogenous hnRNPA1 antibody. The results of each group are expressed as mean ± SD. **P* < 0.05, ***P* < 0.01, ****P* < 0.001. (**B**) Effect of phosphorylation on the subcellular localization of hnRNPA1 was verified by cell fractionation of KYSE150 cells. N represents the nuclear component, characterized by lamin A/C antibody, and C represents the cytoplasmic component, characterized by GAPDH antibody, HA antibody was used to detect the overexpressed hnRNPA1, and C/N represents the grayscale ratio of overexpressed hnRNPA1 in the cytoplasm and nucleus.

# Supplementary Tables

| Supplementary Table S1. UAG sequence mutations in LOXL2 exon 13 | | |
| --- | --- | --- |
| Mutation type | Score | 5′ SS / 3′ SS strength |
| WT-UAG (5′ SS) | 11.87450 |  |
| UAG→~~UAG~~ | 6.82440 | reduced |
| UAG→UAGUAGUAG | 11.33330 | unchanged |
| UAG→UAGUAGGGC | 11.97290 | unchanged |
| UAG→UAC | 7.00550 | reduced |
| UAG→UCG | 9.63790 | reduced |
| UAG→UGG | 9.56900 | reduced |
| UAG→UUG | 9.50080 | reduced |
| UAG→GAG | 12.97180 | increased |
| UAG→CUG | 11.65160 | unchanged |
| UAG→UAGGGC + CUG | 11.75000 | unchanged |
| UAG→CCG | 11.78870 | unchanged |
| UAG→UAGGGC + CCG | 11.88710 | unchanged |
| UAG→UAGGGC + UCG | 9.73630 | weakened |
| UAG→UAGGGC + GAG | 13.07020 | increased |
|  |  |  |
| WT-UAG (3′ SS) | 10.66060 |  |
| UAG→AAG | 8.38050 | reduced |
| UAG→CAG | 12.24890 | increased |
| CUAG→C~~U~~AG | 12.05040 | increased |
| UaggUU→UaggGC | 10.48690 | unchanged |
| CCUCUGCCCUGCUUCU  →CGUCUGCCCUGCUUGU | 9.00230 | reduced |

Underlines indicate insertion, deletion, conversion, or transposition of mutation sites.

| Supplementary Table S5. Plasmids and gene cloning primers used in this paper | |
| --- | --- |
| Plasmid | Primer (5′-3′) |
| pcDNA3 (vector) |  |
| pcDNA3-LOXL2-minigene (L2mini)  NC_000008 | F: CAGTGTGCTGGAATTCACATCCAGAAGAATTACGAGTG |
|  | R: TAGATGCATGCTCGAGCAAGTTTCAGTAAAAACCACAGG |
| pcDNA3-LOXL2-minigene -U13C+A14C (U13C+A14C) | F: CTGGATGTACAACTGCCACACCGGTAAGGCCAGGCCGCC |
|  | R: GGTGTGGCAGTTGTACATCCAGATGCGGTGGCCGTCATA |
| pcDNA3.1-N-SBP-HA (vector-HA) |  |
| pcDNA3.1-N-SBP-HA-HNRNPA1A (hnRNPA1)  NM_002136 | F: CGCCGGAGGTGAATTCTCTAAGTCAGAGTCTCCTAA |
|  | R: GCCCTCTAGACTCGAGTTAAAATCTTCTGCCACTGC |
| pcDNA3.1-N-SBP-HA-HNRNPA1B (hnRNPA1B)  NM_031157 | F1: CGCCGGAGGTGAATTCTCTAAGTCAGAGTCTCCTAA |
|  | R1: CCGTTGTTATAGCTGTCATAGCTGCCACTC |
|  | F2: GAGTGGCAGCTATGACAGCTATAACAACGG |
|  | R2: GCCCTCTAGACTCGAGTTAAAATCTTCTGCCACTGC |
| pcDNA3.1-N-SBP-HA-HNRNPA1-S4/6A (S4/6A) | F: AGGTGAATTCTCTAAGGCAGAGGCTCCTAAAGAGCCCG |
|  | R: CCTCTGCCTTAGAGAATTCACCTCCGGCGTAGTCAGGC |
| pcDNA3.1-N-SBP-HA-HNRNPA1-S4/6D (S4/6D) | F: AGGTGAATTCTCTAAGGATGAGGATCCTAAAGAGCCCGAACAGC |
|  | R: TCCTCATCCTTAGAGAATTCACCTCCGGCGTAGTCAGGCACGT |
| pcDNA3.1-N-SBP-HA-HNRNPA1-S91/95A (S91/95A) | F: CCAAAGAGAGCTGTCGCCAGAGAAGATGCTCAAAGACCAGGTG |
|  | R: CATCTTCTCTGGCGACAGCTCTCTTTGGTTCCACAACTCTTCC |
| pcDNA3.1-N-SBP-HA-HNRNPA1-S91/95D (S91/95D) | F: CCAAAGAGAGCTGTCGACAGAGAAGATGATCAAAGACCAGGTGCCCA |
|  | R: TCATCTTCTCTGTCGACAGCTCTCTTTGGTTCCACAACTCTTCCATC |
| pcDNA3.1-N-SBP-HA-HNRNPA1-S91A (S91A) | F: CCAAAGAGAGCTGTCGCCAGAGAAGATTCTC |
|  | R: CGACAGCTCTCTTTGGTTCCACAACTCTTCC |
| pcDNA3.1-N-SBP-HA-HNRNPA1-S91D (S91D) | F: CCAAAGAGAGCTGTCGACAGAGAAGATTCTCA |
|  | R: TCGACAGCTCTCTTTGGTTCCACAACTCTTCC |
| pcDNA3.1-N-SBP-HA-HNRNPA1-S95A (S95A) | F: GTCTCCAGAGAAGATGCTCAAAGACCAGGTG |
|  | R: CATCTTCTCTGGAGACAGCTCTCTTTGGTTC |
| pcDNA3.1-N-SBP-HA-HNRNPA1-S95D (S95D) | F: GTCTCCAGAGAAGATGATCAAAGACCAGGTGC |
|  | R: TCATCTTCTCTGGAGACAGCTCTCTTTGGTTC |
| pcDNA3.1-N-SBP-HA-HNRNPA1-S91D/95A (S91D/95A) | F: GTCGACAGAGAAGATGCTCAAAGACCAGGTG |
|  | R: CATCTTCTCTGTCGACAGCTCTCTTTGGTTC |
| pcDNA3.1-N-SBP-HA-HNRNPA1-S91A/95D (S91A/95D) | F: GTCGCCAGAGAAGATGATCAAAGACCAGGTGC |
|  | R: TCATCTTCTCTGGCGACAGCTCTCTTTGGTTC |
| pcDNA3.1-N-SBP-HA-HNRNPA1-S309/310A (S309/310A) | F: TGGCTATGGCGGTTCCGCCGCCAGCAGTAGCTATGGC |
|  | R: GCGGCGGAACCGCCATAGCCACCTTGGTTTCGTGGTT |
| pcDNA3.1-N-SBP-HA-HNRNPA1-S309/310D (S309/310D) | F: TGGCTATGGCGGTTCCGACGACAGCAGTAGCTATGGC |
|  | R: TCGTCGGAACCGCCATAGCCACCTTGGTTTCGTGGTT |
| pcDNA3.1-N-SBP-HA-HNRNPA1-S316A (S316A) | F: AGCAGCAGTAGCTATGGCGCTGGCAGAAGATTTTAACT |
|  | R: GCGCCATAGCTACTGCTGCTGCTGGAACCGCCATAGCC |
| pcDNA3.1-N-SBP-HA-HNRNPA1-S316D (S316D) | F: AGCAGCAGTAGCTATGGCGATGGCAGAAGATTTTAACT |
|  | R: TCGCCATAGCTACTGCTGCTGCTGGAACCGCCATAGCC |
| pcDNA3.1-N-SBP-HA-HNRNPA1-ΔM1 (ΔRRM1) | F1: ACCCAAGCTGGCTAGCATGGGAATGGATGAG |
|  | R1: GGCACCTGGCAGCTGTTCGGGCTCTTTAG |
|  | F2: CGAACAGCTGCCAGGTGCCCACTTAACTGT |
|  | R2: GCCCTCTAGACTCGAGTTAAAATCTTCTGCCACTGC |
| pcDNA3.1-N-SBP-HA-HNRNPA1-ΔM2 (ΔRRM2)  pcDNA3.1-N-SBP-HA-HNRNPA1-ΔM1/2 (ΔRRM1/2) | F1: ACCCAAGCTGGCTAGCATGGGAATGGATGAG |
|  | R1: TAGCCATCTCCACAGTTAAGTGGGCACCTG |
|  | F2: CTTAACTGTGGAGATGGCTAGTGCTTCATC |
|  | R2: GCCCTCTAGACTCGAGTTAAAATCTTCTGCCACTGC |
| pcDNA3.1-N-SBP-HA-HNRNPA1-ΔRGG (ΔRGG) | F1: ACCCAAGCTGGCTAGCATGGGAATGGATGAG |
|  | R1: AGCCATCCCCACCGAAGTTGTCATTCCCAC |
|  | F2: CAACTTCGGTGGGGATGGCTATAATGGATT |
|  | R2: GCCCTCTAGACTCGAGTTAAAATCTTCTGCCACTGC |
| pcDNA3.1-N-SBP-HA-PTBP1 (PTBP1)  NM_002819 | F:CGCCGGAGGTGAATTCGACGGCATTGTCCCAGATAT |
|  | R: GCCCTCTAGACTCGAGCTAGATGGTGGACTTGGAGA |
| pcDNA3.1-N-SBP-HA-SRSF1 (SRSF1)  NM_006924.5 | F: CGCCGGAGGTGAATTCTCGGGAGGTGGTGTGATTCG |
|  | R: GCCCTCTAGACTCGAGTTATGTACGAGAGCGAGATCTGC |
| pcDNA3.1-N-SBP-HA-SRSF2 (SRSF2)  NM_001195427 | F: CGCCGGAGGTGAATTCAGCTACGGCCGCCCCCCTC |
|  | R: GCCCTCTAGACTCGAGTTAAGAGGACACCGCTCCTT |
| pcDNA3.1-N-SBP-HA-SRSF5 (SRSF5)  NM_006925.5 | F: CGCCGGAGGTGAATTCAGTGGCTGTCGGGTATTCAT |
|  | R: GCCCTCTAGACTCGAGTTAATTGCCACTGTCAACTG |
| pcDNA3.1-N-SBP-HA-SRSF6 (SRSF6)  NM_006275.6 | F: CGCCGGAGGTGAATTCCCGCGCGTCTACATAGGAC |
|  | R: GCCCTCTAGACTCGAGTTAATCTCTGGAACTCGACCTGG |
| pCMV-N-Flag（vector-Flag) |  |
| pCMV-N-Flag-SRSF3 (SRSF3)  NM_003017 | F: ATGGAGGCCCGAATTCCCCATCGTGATTCCTGTCCATT |
|  | R: GCCGCGGTACCTCGAGCTATTTCCTTTCATTTGACC |
| pCMV-N-Flag-SRSF4 (SRSF4)  NM_005626 | F: ATGGAGGCCCGAATTCCACCGCGGGTGTACATCGGCCG |
|  | R: GCCGCGGTACCTCGAGTTAGGACCTTGAGTGGGACC |
| pCMV-N-Flag-SRSF7 (SRSF7)  NM_001031684 | F: ATGGAGGCCCGAATTCCATCGCGTTACGGGCGGTACGG |
|  | R: GCCGCGGTACCTCGAGTCAGTCCATTCTTTCAGGAC |
| pCMV-N-Flag-SRSF8 (SRSF8)  NM_032102 | F: ATGGAGGCCCGAATTCCAAGCTGCGGCCGCCCCCCTCC |
|  | R: GCCGCGGTACCTCGAGTTAAGAGGACATCTGTCCTT |
| pCMV-N-Flag-SRSF9 (SRSF9)  NM_003769 | F: ATGGAGGCCCGAATTCCATCGGGCTGGGCGGACGAGCG |
|  | R: GCCGCGGTACCTCGAGTCAGTAGGGCCTGAAAGGAG |
| pCMV-N-Flag-SRSF10 (SRSF10)  NM_054016.4 | F: ATGGAGGCCCGAATTCCATCCCGCTACCTGCGTCCCCC |
|  | R: GCCGCGGTACCTCGAGTCAGTGGCCACTGGACTTAG |
| pCMV-N-Flag-SRSF11 (SRSF11)  NM_004768 | F: ATGGAGGCCCGAATTCCAAGCAACACTACCGTCGTCCC |
|  | R: GCCGCGGTACCTCGAGTCAGTCACTCATATCCATGT |
| pCMV-N-Flag-SRSF12V1 (SRSF12V1)  NM_080743 | F: ATGGAGGCCCGAATTCCATCTCGCTACACGAGGCCCCC |
|  | R: GCCGCGGTACCTCGAGTCACCAACTGTTTTTATGAC |
| pCMV-N-Flag-SRSF12V2 (SRSF12V2)  NM_001376896 | F: ATGGAGGCCCGAATTCCCAAATCAAAAGAACGTCATCC |
|  | R: GCCGCGGTACCTCGAGTCACCAACTGTTTTTATGAC |
| pCMV-N-Flag-HNRNPA1 (Flag-HNRNPA1) | F: ATGGAGGCCCGAATTCCCTCTAAGTCAGAGTCTCCTAA |
|  | R: GCCGCGGTACCTCGAGTTAAAATCTTCTGCCACTGC |
| pCMV-N-Flag-HNRNPA1-S91/95D (Flag-S91/95D) | F: ATGGAGGCCCGAATTCCCTCTAAGTCAGAGTCTCCTAA |
|  | R: GCCGCGGTACCTCGAGTTAAAATCTTCTGCCACTGC |
| pCMV-N-Flag-HNRNPA1-ΔRRM1 (Flag-ΔRRM1) | F: ATGGAGGCCCGAATTCCCTCTAAGTCAGAGTCTCCTAA |
|  | R: GCCGCGGTACCTCGAGTTAAAATCTTCTGCCACTGC |
| pCMV-N-Flag-HNRNPA1-ΔRRM2 (Flag-ΔRRM2) | F: ATGGAGGCCCGAATTCCCTCTAAGTCAGAGTCTCCTAA |
|  | R: GCCGCGGTACCTCGAGTTAAAATCTTCTGCCACTGC |
| pCMV-N-Flag-HNRNPA1-ΔRRM1/2 (Flag-ΔRRM1/2) | F: ATGGAGGCCCGAATTCCCTCTAAGTCAGAGTCTCCTAA |
|  | R: GCCGCGGTACCTCGAGTTAAAATCTTCTGCCACTGC |
| pCMV-N-Flag-HNRNPA1-ΔRGG (Flag-ΔRGG) | F: ATGGAGGCCCGAATTCCCTCTAAGTCAGAGTCTCCTAA |
|  | R: GCCGCGGTACCTCGAGTTAAAATCTTCTGCCACTGC |
| pET-32a-His |  |
| HNRNPA1A-His-F | F: GGCTGATATCGGATCCTCTAAGTCAGAGTCTCCTAAAGAGCCC |
| HNRNPA1A-His-R | R: GGTGGTGGTGCTCGAGAAATCTTCTGCCACTGCCAT |

The name in parentheses is the abbreviation in the experimental results graph of this article. The NC/NM number is a gene or transcript amplified by PCR.

| Supplementary Table S6. Additional primers | |
| --- | --- |
| Primer | Sequence (5′-3′) |
| T7 | TTAATACGACTCACTATAGGG |
| BGH | TAGAAGGCACAGTCGAGG |
| LOXL2-minigene-test-R-2 | GACAGCTGGTTGTTTAAGAG |
| TREfor | TCCACGCTGTTTTGACCTCC |
| EBVrev | GTGGTTTGTCCAAACTCATC |
| HNRNPA1-qF | GCTAGTGCTTCATCCAGCCA |
| HNRNPA1-qR | CTCCACGACCGAAGTTGTCA |
| SRSF9-qF | ATATGCCCTGCGTAAACTGG |
| SRSF9-qR | AGGGCCTGAAAGGAGAGAAG |
| SRSF10-qF | AGTGGATTTGTGGACGGCAG |
| SRSF10-qR | GACCGACTTCTTGATCTCCTCC |
| SRSF12V1-qF | GCCGTGAGTTTGGTCGATATG |
| SRSF12V1-qR | GACGGCCACATACCCACTT |
| βactin-qF | CAACTGGGACGACATGGAGAAA |
| βactin-qR | GATAGCAACGTACATGGCTGGG |
| LOXL2-RIP-test-F | TGGAGGTGGCCGCAGAGGGAACAAGCA |
| LOXL2-RIP-test-R | TTAGGAAGCTGCCTCTGGGTCCTTGAGAAA |
| LOXL2-F | GGACATGTACCGCCATGACA |
| LOXL2WT-qR | ATAGCGGCTCCTGCATTTCA |
| LOXL2e13-qR | GCTGAAGGAACCACCTGGAAC |

| Supplementary Table S7. The siRNAs used in this paper | |
| --- | --- |
| Name | Sequences (5′-3′) |
| siHNRNPA1#1 | CAGCUGAGGAAGCUCUUCATT |
| siHNRNPA1#4 | AGAUAUUUGUUGGUGGCAUUATT |

| Supplementary Table S8. Biotinylated RNAs used in this paper | |
| --- | --- |
| Name | Sequences (5′-3′ biotin) |
| Oligo 1 | AGCAGCCUCCUCUGCCCUGCUUCUCCCUAGGUUGUU |
| Oligo 2 | ACAACUGCCACAUAGGUAAGGCCAGGCCGC |
| Oligo 2-U13C+A14C (U13C+A14C) | ACAACUGCCACACCGGUAAGGCCAGGCCGC |
